# Effects of meteorological factors on influenza transmissibility by virus type/subtype

**DOI:** 10.1186/s12889-024-17961-9

**Published:** 2024-02-16

**Authors:** Ze-Lin Yan, Wen-Hui Liu, Yu-Xiang Long, Bo-Wen Ming, Zhou Yang, Peng-Zhe Qin, Chun-Quan Ou, Li Li

**Affiliations:** 1 State Key Laboratory of Organ Failure Research, Department of Biostatistics, Guangdong Provincial Key Laboratory of Tropical Disease Research, School of Public Health, Southern Medical University, Guangzhou, Guangdong China; 2 Guangzhou Center for Disease Control and Prevention, Guangzhou, Guangdong China

**Keywords:** Meteorological factors, Hourly temperature variability, Influenza, Instantaneous effective reproductive  number, Distributed lag non-linear model

## Abstract

**Background:**

Quantitative evidence on the impact of meteorological factors on influenza transmissibility across different virus types/subtypes is scarce, and no previous studies have reported the effect of hourly temperature variability (HTV) on influenza transmissibility. Herein, we explored the associations between meteorological factors and influenza transmissibility according to the influenza type and subtype in Guangzhou, a subtropical city in China.

**Methods:**

We collected influenza surveillance and meteorological data of Guangzhou between October 2010 and December 2019. Influenza transmissibility was measured using the instantaneous effective reproductive number (*R*_*t*_). A gamma regression with a log link combined with a distributed lag non-linear model was used to assess the associations of daily meteorological factors with *R*_*t*_ by influenza types/subtypes.

**Results:**

The exposure-response relationship between ambient temperature and *R*_*t*_ was non-linear, with elevated transmissibility at low and high temperatures. Influenza transmissibility increased as HTV increased when HTV < around 4.5 °C. A non-linear association was observed between absolute humidity and *R*_*t*_, with increased transmissibility at low absolute humidity and at around 19 g/m^3^. Relative humidity had a U-shaped association with influenza transmissibility. The associations between meteorological factors and influenza transmissibility varied according to the influenza type and subtype: elevated transmissibility was observed at high ambient temperatures for influenza A(H3N2), but not for influenza A(H1N1)pdm09; transmissibility of influenza A(H1N1)pdm09 increased as HTV increased when HTV < around 4.5 °C, but the transmissibility decreased with HTV when HTV < 2.5 °C and 3.0 °C for influenza A(H3N2) and B, respectively; positive association of *R*_*t*_ with absolute humidity was witnessed for influenza A(H3N2) even when absolute humidity was larger than 19 g/m^3^, which was different from that for influenza A(H1N1)pdm09 and influenza B.

**Conclusions:**

Temperature variability has an impact on influenza transmissibility. Ambient temperature, temperature variability, and humidity influence the transmissibility of different influenza types/subtypes discrepantly. Our findings have important implications for improving preparedness for influenza epidemics, especially under climate change conditions.

**Supplementary Information:**

The online version contains supplementary material available at 10.1186/s12889-024-17961-9.

## Introduction

Influenza is a significant threat to public health worldwide. Each year, seasonal influenza causes approximately three to 5 million cases of severe illness and 290,000–650,000 respiratory deaths globally [1]. Influenza seasonality varies across climatic zones. Specifically, influenza commonly peaks during winter in temperate zones [2], whereas its seasonality is more complicated and difficult to predict in subtropical and tropical regions with semi-annual peaks or year-round activity [3].

Understanding the drivers of influenza transmission will inform preventive and control measures. Previous studies have attempted to reveal the potential role of meteorological factors (e.g., ambient temperature and humidity) and school closure in modulating influenza transmission [4–8]. However, different findings on the association between meteorological variables and influenza transmissibility have been reported in previous studies [5–8]. For example, Zhang et al. detected increased influenza transmissibility at both low and high ambient temperatures in China [7], whereas Zhang et al. did not observe increased transmissibility at high temperature in Guangzhou, China [8]. Regarding the association between outdoor absolute humidity and influenza transmissibility, U-shaped [5], non-linear inverse [8], and statistically non-significant associations [6] have been reported previously. Different study locations and study periods are likely to contribute to the inconclusive findings. Differential influenza types/subtypes predominate at different locations and time periods of diverse meteorological characteristics and have discrepant transmissibility. Therefore, the influence of meteorological factors on influenza transmissibility is expected to vary according to the influenza type and subtype. Further determination of the meteorological drivers of influenza transmission by type/subtype would have important implications for precise interventions against influenza. Nevertheless, only one study has reported the type/subtype-specific impact of meteorological factors on influenza transmissibility [7].

Under climate change, extreme weather events will occur more frequently in the future, and temperatures are likely to become more unstable. It has been projected that each degree of global warming will result in a 10% increase in temperature variability in the subtropical hotspots of the Northern Hemisphere in the future [9]. Exposure to temperature variability is inevitable. It is hypothesized that large temperature variability is likely to facilitate the transmission of influenza. However, only one study has investigated the impact of short-term temperature variability on influenza transmission: Zhang et al. has reported a U-shaped association between the diurnal temperature range (DTR) and influenza transmissibility [8]. DTR is an index that only accounts for the variation in extreme temperatures within a day and does not consider the inter-day variation in temperatures. Further efforts are warranted to investigate the impact of temperature variability on influenza transmissibility using an index that accounts for both intra- and inter-day variations. This includes the hourly temperature variability (HTV; the standard deviation of hourly temperatures), which has been commonly used to assess the impact of temperature variability on health outcomes, such as hospital admissions and mortality [10–12].

Guangzhou is the largest city in the south of China (latitude: 23°07′N; longitude 113°15′E) and has a population density of 2059 per km^2^ in 2019. Meanwhile, Guangzhou is a transportation hub, the risk of influenza transmission is high. In this study, we explored the effects of meteorological factors (i.e., ambient mean temperature, HTV, absolute humidity, and relative humidity) on influenza transmissibility by type/subtype in Guangzhou, China.

## Methods

### Data collection

Influenza surveillance data were obtained from the Guangzhou Center for Disease Control and Prevention (CDC). The week in which the first Monday of January lied was defined as the start week of a specific year. We extracted the weekly fraction of consultations for influenza-like illness (ILI: body temperature ≥ 38 °C with cough or sore throat) among outpatient visits at sentinel hospitals in Guangzhou between October 4, 2010 (the first Monday of October 2010) and January 5, 2020 (the end of the last week of 2019). In addition, virological data, that is, weekly percentages of specimens that tested positive for influenza A(H1N1)pdm09, A(H3N2), and B, were also compiled.

We obtained hourly data on temperature recorded at two meters above the land surface from the fifth generation of European Reanalysis Land (ERA5-Land) dataset at a spatial resolution of 0.1° × 0.1° (~ 9 km × 9 km) [13]. The hourly relative humidity (for 06:00, 09:00, 12:00, 15:00, and 18:00) at a height of two meters above the surface were collected from Agrometeorological ERA5 with a spatial resolution of 0.1° × 0.1° as well [14]. We averaged data points of all of the 89 grids to obtain the hour data for the subsequent analysis. And the daily mean temperature and relative humidity were obtained by averaging the hourly data. Information on holiday-related school closures, including public holidays, weekends, winter holidays, and summer holidays, were also collected. The Public Security Bureau of Guangzhou Municipality provided data on annual population size.

### Calculation of influenza virus activity proxy

To determine influenza virus activity, the weekly ILI+ was calculated by multiplying the ILI consultation rate (ILI%, the proportion of patients with ILI among the outpatients) by the rate of specimens positive for influenza (lab%) [5, 7, 15]. ILI+ would be an ideal measure of the incidence of influenza infection under some conditions, e.g. (1) the proportion of ILIs that seek medical consultations is stable over time; (2) the sample from sentinel hospitals for ILI% estimation is representative of the study population; (3) the sample of viral testing for lab% estimation is representative of the medical consultations for ILI; (4) the performance of diagnosis method for influenza virus does not change over time [16].

### Estimation of the daily instantaneous effective reproductive number

The weekly number of influenza infections was estimated as ILI+ multiplied by the population size and a conversion rate (*γ* = 1) [17]. The daily number of influenza infections was interpolated using the spline function [4]. Influenza transmissibility was measured using the instantaneous effective reproductive number (*R*_*t*_), defined as the average number of secondary infections resulting from an infectious individual at time *t*. We estimated *R*_*t*_ as the number of new infections at time *t* (i.e. *I*_*t*_) divided by the total infectiousness of infected individual at time *t* [18]:

$${R}_t=\frac{I_t}{\sum_{s=1}^t{I}_{t-s}{w}_s}$$

where *w*_*s*_ is the current infectiousness of individuals which were infected *s* days ago, based on the distribution of serial interval. We assumed that the serial intervals followed gamma distributions with means ± standard deviations of 3.3 ± 1.7, 3.08 ± 1.39, 3.48 ± 1.88, and 3.72 ± 1.95 for influenza, A(H1N1)pdm09, A(H3N2), and B, respectively [19]. The estimates of *R*_*t*_ can be highly fluctuating due to small time step of data. To address this issue, we estimated the *R*_*t*_ over a 7-day time window, assuming that the daily *R*_*t*_ did not change over this time window [8].

### Assessment of the associations between meteorological factors and *R*_*t*_

Our preliminary analysis suggested that *R*_*t*_ fitted the gamma distribution better than the lognormal distribution in terms of Akaike’s Information Criteria. Therefore, a gamma regression with a log link combined with a distributed lag non-linear model was used to assess the potentially non-linear effects of meteorological factors on influenza transmissibility [20], after adjusting for inter-epidemic effects and the effects of depletion of susceptibility over time and holidays. The analysis was restricted to the data from maximum of 9 weeks either side of the peak of influenza epidemic (Additional file 1) to prevent the potential impact of the low and irregular reporting at the very start and end of each epidemic. HTV was not included in the regression model when the effect of temperature was assessed as it may mediate the effect of temperature on influenza transmissibility. Temperature and absolute humidity were commonly highly correlated; therefore, these two variables were not included in the same model when analyzing the effect of each of these variables on influenza transmissibility. Absolute humidity and relative humidity were not included in the same model. Details of the models fitted are provided in Additional file 1. Wald test was applied to assess the statistical significance of each meteorological factor.

Sensitivity analyses were conducted to assess the robustness of results by (1) setting *γ* = 0.5, *γ* = 0.05, *γ* = 0.005; (2) changing the mean ± standard deviation of serial interval for influenza and different influenza types/subtypes to 3.3 ± 1.7 and 2.6 ± 1.5 [21]; (3) considering data from maximum 8–10 weeks either side of each epidemic peak. All statistical analyses were performed using the R software (version 4.1.1; R Foundation for Statistical Computing).

## Results

**Fig. 1:**
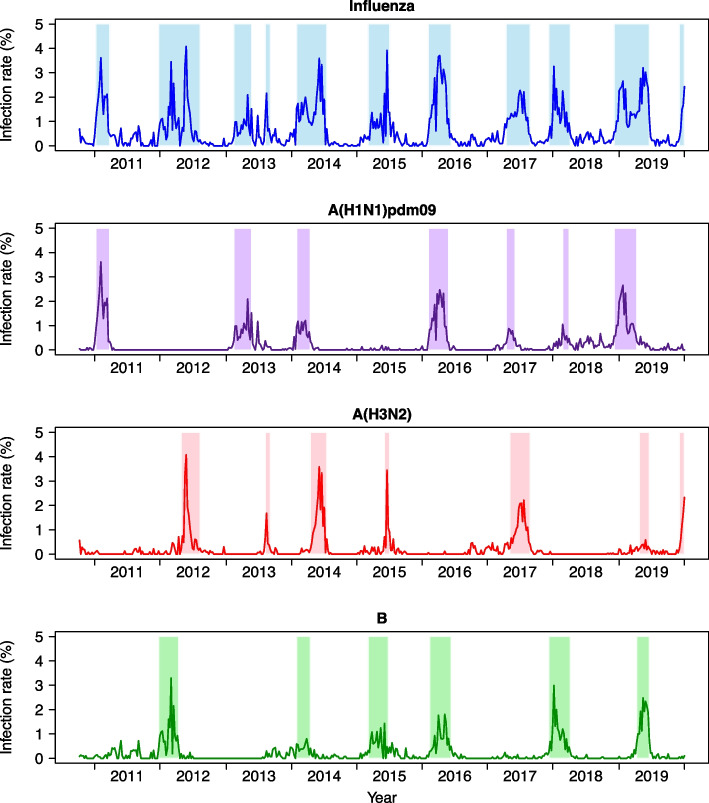
Weekly influenza infection rates in Guangzhou, China from October 2010 to December 2019. Lines indicate the influenza infection rate; colored areas, influenza epidemics used in the gamma regression analysis

A total of 11 influenza epidemics were detected during the study period (Fig. 1). Influenza epidemics varied by influenza type/subtype. Specifically, there were seven, seven, and six influenza A(H1N1)pdm09, A(H3N2), and B epidemics, respectively, with different lengths and peaks. Influenza A(H1N1)pdm09 and A(H3N2) co-circulated in 6/200 epidemic weeks (200 epidemic weeks were considered in the regression analysis of the associations between meteorological factors and *R*_*t*_), influenza A(H3N2) and B co-circulated in 14/200 epidemic weeks, and influenza A(H1N1)pdm09 and B co-circulated in 35/200 epidemic weeks. We estimated that an infected individual could cause a median of 1.007 secondary cases (Table 1 and Additional file 1). The median *R*_*t*_ of influenza A(H3N2) (1.049) was higher than that of influenza A(H1N1)pdm09 (1.009) and influenza B (1.004) (Table 1 and Additional file 1).

**Table 1:** Summary statistics of the estimates of daily instantaneous effective reproductive number in Guangzhou, China

Influenza epidemics	Mean	*SD*	Minimum	*P*_25_	Median	*P*_75_	Maximum
All influenza	1.044	0.379	< 0.001	0.836	1.007	1.185	4.129
A(H1N1)pdm09	1.017	0.287	0.080	0.855	1.009	1.171	2.543
A(H3N2)	1.086	0.477	0.072	0.778	1.049	1.251	3.935
B	1.087	0.610	< 0.001	0.769	1.004	1.246	7.920

Abbreviations: *SD* standard deviation, *P*_*25*_ the 25th percentile, *P*_*75*_ the 75th percentile

**Fig. 2:**
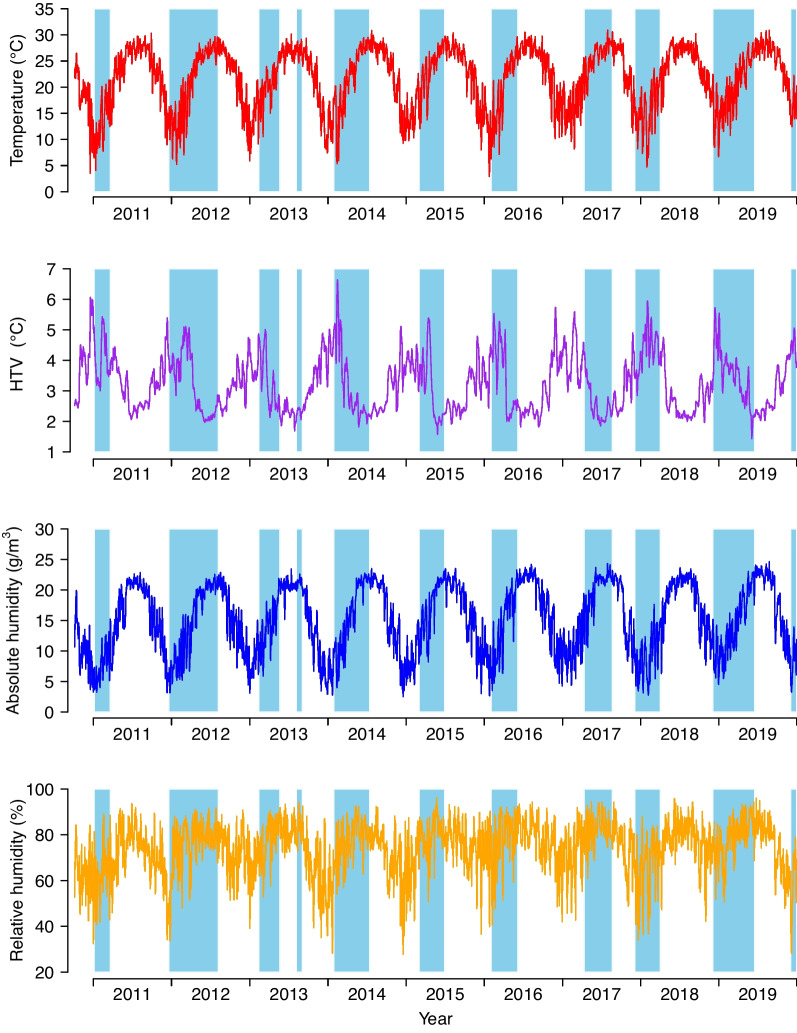
Time-series of daily meteorological factors in Guangzhou, China from October 4, 2010 to December 30, 2019. Lines indicate the time-series of daily meteorological factors and colored areas, influenza epidemics used in the gamma regression analysis. Abbreviation: HTV, hourly temperature variability

During the influenza epidemics, the mean daily temperature, HTV over 0–14 days, daily absolute humidity, and relative humidity were 20.74 °C, 3.34 °C, 14.78 g/m^3^, and 76.06%, respectively (Table 2 and Fig. 2). On average, temperature and absolute humidity were higher during influenza A(H3N2) epidemics than during other epidemics, whereas the mean HTV was lower during influenza A(H3N2) epidemics than during other influenza epidemics.

**Table 2:** Summary statistics of meteorological factors in different influenza epidemics in Guangzhou, China

Variables	Influenza epidemics	Mean	*SD*	Minimum	*P*_25_	Median	*P*_75_	Maximum
Temperature (°C)	All influenza	20.74	6.07	4.08	15.85	21.86	25.87	30.92
	A(H1N1)pdm09	18.50	5.22	4.08	14.75	19.46	22.65	28.16
	A(H3N2)	25.90	3.19	12.45	25.04	26.68	27.89	30.92
	B	19.40	5.89	4.72	14.80	20.35	24.39	29.66
HTV (°C)	All influenza	3.34	1.05	1.43	2.37	3.25	4.13	6.64
	A(H1N1)pdm09	3.74	0.96	1.94	3.06	3.76	4.41	6.64
	A(H3N2)	2.48	0.60	1.43	2.14	2.31	2.59	5.06
	B	3.64	1.07	1.43	2.67	3.74	4.46	6.64
Absolute humidity (g/m^3^)	All influenza	14.78	5.74	2.75	9.78	15.17	20.28	24.31
	A(H1N1)pdm09	12.55	4.60	3.23	8.74	12.51	16.17	23.68
	A(H3N2)	19.81	3.56	3.25	19.20	21.05	21.81	24.31
	B	13.52	5.48	2.75	8.73	13.56	18.24	24.05
Relative humidity (%)	All influenza	76.06	11.47	28.10	70.60	78.49	84.22	96.14
	A(H1N1)pdm09	74.72	10.72	40.57	68.56	76.24	83.11	94.47
	A(H3N2)	80.39	8.76	28.10	77.13	80.91	85.80	94.47
	B	75.22	12.26	33.58	68.97	77.89	84.35	96.14

Abbreviations: *SD* standard deviation, *P*_*25*_ the 25th percentile, *P*_*75*_ the 75th percentile, *HTV* hourly temperature variability

**Fig. 3:**
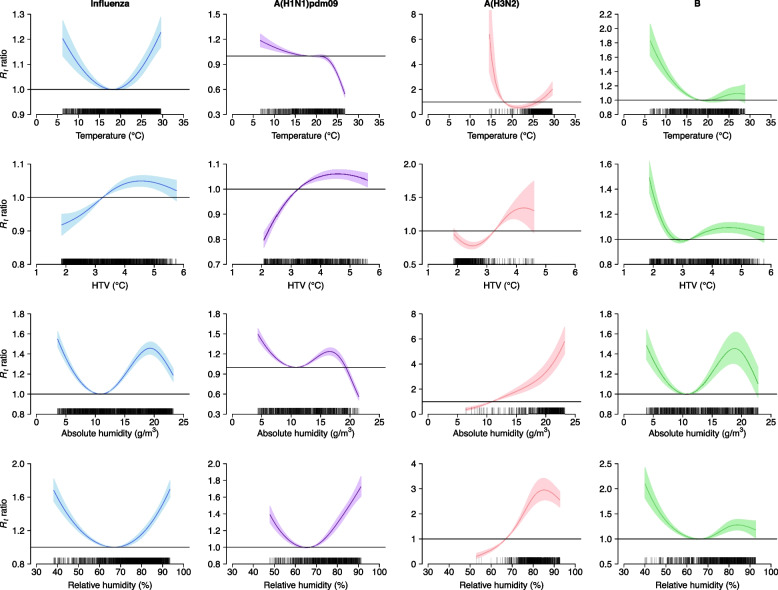
Exposure-response curves of the associations of daily instantaneous effective reproductive number (*R*_*t*_) with various meteorological variables. Curves and colored areas represent the point estimates of *R*_*t*_ ratios and the corresponding confidence intervals, respectively. The ticks along the x-axis are observed meteorological data. Horizontal lines indicating *R*_*t*_ ratio = 1 were also plotted. The *R*_*t*_ ratios are the ratio of predicted *R*_*t*_ with respect to reference values for the meteorological factors of mean temperature, hourly temperature variability (HTV), absolute humidity, and relative humidity set to 18.12 °C, 3.25 °C, 10.88 g/m^3^, and 66.99%, respectively. Reference values for the mean temperature, absolute humidity, and relative humidity corresponded to the lowest transmission risk of influenza, and the median is used as the reference value for HTV. We depicted the associations excluding the 10 lowest and the 10 largest values of meteorological factors, avoiding the potentially unrobust estimates due to small sample size

Figure 3 shows the exposure-response associations between meteorological factors and *R*_*t*_. And Additional file 1 present the results of Wald tests of the statistical significance of each meteorological factor. It was apparent that the association between ambient temperature and *R*_*t*_ was non-linear, with elevated transmissibility at low and high temperatures. Influenza transmissibility increased as HTV increased when HTV < around 4.5 °C. A non-linear association was observed between absolute humidity and *R*_*t*_, with increased transmissibility at low absolute humidity and at around 19 g/m^3^. Relative humidity had a U-shaped association with influenza transmissibility.

As expected, the associations between meteorological factors and influenza transmissibility varied according to the influenza type and subtype (Fig. 3 and Additional file 1). Regarding the association with ambient temperature, the *R*_*t*_ ratio for influenza A(H1N1)pdm09 in large decreased with temperature and the *R*_*t*_ ratio for influenza B declined with temperature when it was colder than around 19 °C, whereas, the lowest *R*_*t*_ ratio for influenza A(H3N2) was witnessed at around 21 °C, higher than which, the *R*_*t*_ ratio increased with temperature. As for the relationship with HTV, the *R*_*t*_ ratio for influenza A(H1N1)pdm09 increased with HTV when HTV < around 4.5 °C, while the lowest *R*_*t*_ ratio for influenza A(H3N2) was observed at 2.5 °C and the transmissibility of influenza B decreased with HTV when HTV < 3.0 °C. An initially decreasing, then increasing, and finally declining trend was observed in the associations between absolute humidity and transmissibility of influenza A(H1N1)pdm09 and influenza B. For influenza A(H3N2), the *R*_*t*_ ratio increased with absolute humidity.

Point estimates of *R*_*t*_ were robust to the change in the conversion rate, although the 95% credible intervals of *R*_*t*_ were wider when lower conversion rates were used to estimate the number of influenza infections (Additional file 1). *R*_*t*_ estimates were similar to the results of main analysis when assuming that mean ± standard deviation of serial interval was 3.3 ± 1.7 (Additional file 1). Both of mean and standard deviation of *R*_*t*_ estimates decreased when assuming that mean ± standard deviation of serial interval was 2.6 ± 1.5 (Additional file 1). The estimated associations of *R*_*t*_ with ambient temperature, HTV, absolute humidity, and relative humidity were in large robust to the conversation rate and to the mean and standard deviation of the serial interval (Additional file 1). Overall, the estimates of the associations between meteorological factors and *R*_*t*_ did not vary substantially when altering the maximum weeks either side of the epidemic peak, although the association between HTV and transmissibility for influenza A(H3N2) was inconclusive (Additional file 1).

## Discussion

This study explored the associations between influenza transmissibility and meteorological variables by influenza type/subtype in Guangzhou, a subtropical city in China. The results showed that influenza transmissibility had a non-liner relationship with ambient temperature, HTV, absolute humidity, and relative humidity, and this relationship differed according to influenza type and subtype.

Ambient temperature and humidity play potential roles in the influenza transmission [22, 23]. We observed that influenza transmissibility increased at both low and high temperatures, consistent with a previous study in China based on the data of 30 provincial-level administrative divisions (PLADs) during 2010–2017 [7]. However, Zhang et al. reported an elevated influenza transmissibility at low temperatures but not at high temperatures in Guangzhou, China during 2005–2021 [8]. We found that relative humidity had a U-shaped association with influenza transmissibility, in accordance with a previous report [8]. In this study, the effect of absolute humidity on influenza transmissibility was relatively complicated, with a high transmission risk occurring at low absolute humidity and at approximately 19 g/m^3^. Zhang et al. reported a similar result in China [7], while Ali et al. identified a U-shaped association in China based on data from nine PLADs [5]. Zhang et al. found that influenza transmissibility decreased with absolute humidity in Guangzhou, China [8]. Lei et al. showed that the association was statistically non-significant in temperate and subtropical regions in China based on data from five PLADs during 2013–2019 [6]. Different study locations, study periods (with differentially predominant circulating influenza types/subtypes and epidemic intensities), and influenza incidences used to estimate *R*_*t*_ (reported number of symptomatic influenza cases or the one estimated based on ILI+) may have contributed to the differential findings on the associations between meteorological variables and influenza transmissibility (Additional file 1).

To the best of our knowledge, this is the first study to reveal the association between HTV and influenza transmissibility. We found that the HTV was positively associated with *R*_*t*_ ratio for influenza when HTV < around 4.5 °C. Similarly, previous studies reported a positive association between DTR and influenza incidence [24] and respiratory infections [25, 26]. Zhang et al. showed a U-shaped curve for the association between DTR and influenza transmissibility [8]. DTR only reflects intra-day variations based on extreme temperatures, whereas HTV reflects both intra- and inter-day variations, not merely based on extreme temperatures. Thus, it is possible that the association between HTV and influenza transmissibility was different from that between DTR and influenza transmissibility, in addition to the reasons mentioned above for the different findings regarding the associations of *R*_*t*_ with ambient temperature and humidity.

The potential impact of meteorological factors on influenza transmissibility can be explained by (1) virus stability and shedding, (2) adaptive immune responses, and (3) changes in human behavior (e.g., spending more time indoors) in response to meteorological factors. Several plausible reasons have been proposed for the high transmissibility at low and high temperatures. First, cold air weakens the nasal mucociliary clearance, facilitates the ordering of lipids on the viral membrane, and strengthens viral stability and viral shedding, thus reinforcing viral amplification and transmission [27, 28]. Second, adaptive immune responses are hindered at low and high temperatures [29, 30], making people more susceptible to influenza infection. Third, people tend to spend more time indoor at low and high temperatures (cooling with air conditioner), leading to more human-to-human contact and increased transmissibility [31]. Regarding the mechanisms of the association between absolute humidity and influenza transmissibility, at low humidity, there could be serious impairments in mucociliary clearance and airway tissue repair mechanisms. Further, global type I interferon-stimulated gene expression could be inhibited following intranasal influenza virus infection [32]. Increased absolute humidity in summer may also result in elevated influenza transmissibility through human behavioral changes. Large temperature variations often occur with sudden changes in temperature. Our findings regarding the positive association between HTV and influenza transmissibility when HTV < around 4.5 °C can be explained by the following possible mechanisms. First, sudden temperature changes cause a more significant inflammatory nasal response, resulting in decreased ability of the nasal cavity to clear the respiratory virus [33]. Second, large temperature variations can affect humoral and cellular immunity [34]. A previous study related sudden temperature changes with an increase in the release of inflammatory mediators associated with mast cells [35]. Increased HTV may have led to a decline in immunity to influenza and influenza transmissibility. Influenza is often transmitted indoors, and there are discrepancies between indoor and outdoor environmental factors such as temperature and humidity [6]. Therefore, indoor-outdoor differences should be considered when interpreting the effects of meteorological factors on influenza transmissibility.

Interestingly, we observed subtype-dependence effects of meteorological factors on influenza transmissibility: high ambient temperatures were associated with elevated transmissibility for influenza A(H3N2), but not for influenza A(H1N1)pdm09; when absolute humidity was larger than around 19 g/m^3^, absolute humidity was still positively associated with transmissibility for influenza A(H3N2), but not for other influenza type/subtype (Fig. 3). The differences in the effects of meteorological factors on influenza transmissibility across influenza types/subtypes may be attributed to disparities in viral properties and efficiency in response to environmental factors [36–38]. Viral inactivation depends on the thermal denaturation of proteins and nucleic acids [39]. The environment may influence the stability of influenza viruses by inducing conformational changes in surface glycoproteins or increasing the ordering of lipids in the viral envelope [28, 40]. Disparities in envelope proteins may be responsible for the differential environmental dependence among influenza types/subtypes [7]. An experimental study also reported strain-dependent variations in the longevity of influenza A(H1N1)pdm09, A(H3N2), and B lineages in droplets [37]. In addition, different influenza types/subtypes predominated in distinct time periods and influenza activity of a specific type/subtype was low in some meteorological conditions, probably resulting in insufficient sample size for the inference of the association between a specific meteorological factor and *R*_*t*_ in these conditions. Further studies are warranted to reveal the mechanisms underlying the different effects of meteorological factors on influenza transmissibility according to influenza type and subtype.

This study has some limitations. First, we did not have data on “true” influenza incidence because a certain proportion of influenza infections did not seek hospital consultation. Therefore, the number of influenza cases reported in surveillance systems is an underestimate of influenza infection. Here, we estimated influenza incidence based on ILI+, as previous studies have suggested that ILI+ may be a better proxy than ILI and virological data for assessing influenza virus activity [16, 41]. We did not consider the delays from infection to case report when inferring the influenza incidence. Further studies are warranted to deal with this issue with an appropriate approach which is robust even when the distribution of the delay from infection to case report is uncertain. Second, we did not examine the associations between meteorological factors and influenza transmissibility by different age groups because the surveillance was designed to delineate the pattern for the whole population and not for different age groups. Third, we focused only on the impact of meteorological factors on influenza transmissibility in a subtropical city. Such efforts would provide invaluable data on the mechanisms of influenza seasonality in subtropical cities, which are not fully understood. More efforts are needed to reveal the mechanisms of influenza seasonality using data from more cities across different climatic zones.

## Conclusions

Temperature variability has an impact on influenza transmissibility. Ambient temperature, temperature variability, and humidity influence the transmissibility of different influenza types/subtypes discrepantly. Our findings have important implications for improving preparedness for influenza epidemics, especially under climate change conditions.

### Supplementary Information

**Additional file 1. **Definition of influenza epidemics. Models. **Fig. S1.** Estimates of daily instantaneous effective reproductive number (R_*t*_) for each influenza epidemic used in the gamma regression analysis. **Fig. S2.** Estimates of daily instantaneous effective reproductive number (R_*t*_) for each influenza A(H1N1)pdm09 epidemic used in the gamma regression analysis. **Fig. S3.** Estimates of daily instantaneous effective reproductive number (R_*t*_) for each influenza A(H3N2) epidemic used in the gamma regression analysis. **Fig. S4.** Estimates of daily instantaneous effective reproductive number (R_*t*_) for each influenza B epidemic used in the gamma regression analysis. **Table S1.** Results of Wald tests of the statistical significance of each meteorological factor. **Table S2.** Summary statistics of the estimates of daily instantaneous effective reproductive number when using different conversion rates to calculate influenza incidence. **Fig. S5.** Estimates of daily instantaneous effective reproductive number (R_*t*_) for each influenza epidemic when setting the conversion rate to 0.5. **Fig. S6.** Estimates of daily instantaneous effective reproductive reproduction number (R_*t*_) for each influenza A(H1N1)pdm09 epidemic when setting the conversion rate to 0.5. **Fig. S7.** Estimates of daily instantaneous effective reproductive number (R_*t*_) for each influenza A(H3N2) epidemic when setting the conversion rate to 0.5. **Fig. S8.** Estimates of daily instantaneous effective reproductive number (R_*t*_) for each influenza B epidemic when setting the conversion rate to 0.5. **Fig. S9.** Estimates of daily instantaneous effective reproductive number (R_*t*_) for each influenza epidemic when setting the conversion rate to 0.05. **Fig. S10.** Estimates of daily instantaneous effective reproductive number (R_*t*_) for each influenza A(H1N1)pdm09 epidemic when setting the conversion rate to 0.05. **Fig. S11.** Estimates of daily instantaneous effective reproductive number (R_*t*_) for each influenza A(H3N2) epidemic when setting the conversion rate to 0.05. **Fig. S12.** Estimates of daily instantaneous effective reproductive number (R_*t*_) for each influenza B epidemic when setting the conversion rate to 0.05. **Fig. S13.** Estimates of daily instantaneous effective reproductive number (R*t*) for each influenza epidemic when setting the conversion rate to 0.005. **Fig. S14.** Estimates of daily instantaneous effective reproductive number (R_*t*_) for each influenza A(H1N1)pdm09 epidemic when setting the conversion rate to 0.005. **Fig. S15.** Estimates of daily instantaneous effective reproductive number (R_*t*_) for each influenza A(H3N2) epidemic when setting the conversion rate to 0.005. **Fig. S16.** Estimates of daily instantaneous effective reproductive number (R_*t*_) for each influenza B epidemic when setting the conversion rate to 0.005. **Table S3.** Summary statistics of the estimates of daily instantaneous effective reproductive number when assuming different means and standard deviations of the serial interval. **Fig. S17.** Estimates of daily instantaneous effective reproductive number (R_*t*_) for each influenza A(H1N1)pdm09 epidemic when assuming the mean and standard deviation of serial interval were 3.3 and 1.7, respectively. **Fig. S18.** Estimates of daily instantaneous effective reproductive number (R_*t*_) for each influenza A(H3N2) epidemic when assuming the mean and standard deviation of serial interval were 3.3 and 1.7, respectively. **Fig. S19.** Estimates of daily instantaneous effective reproductive number (R_*t*_) for each influenza B epidemic when assuming the mean and standard deviation of serial interval were 3.3 and 1.7, respectively. **Fig. S20.** Estimates of daily instantaneous effective reproductive number (R_*t*_) for each influenza epidemic when assuming the mean and standard deviation of serial interval were 2.6 and 1.5, respectively. **Fig. S21.** Estimates of daily instantaneous effective reproductive number (R_*t*_) for each influenza A(H1N1)pdm09 epidemic when assuming the mean and standard deviation of serial interval were 2.6 and 1.5, respectively. **Fig. S22.** Estimates of daily instantaneous effective reproductive number (R_*t*_) for each influenza A(H3N2) epidemic when assuming the mean and standard deviation of serial interval were 2.6 and 1.5, respectively. **Fig. S23.** Estimates of daily instantaneous effective reproductive number (R_*t*_) for each influenza B epidemic when assuming the mean and standard deviation of serial interval were 2.6 and 1.5, respectively. **Fig. S24.** Exposure-response curves of the associations of daily instantaneous effective reproductive number (R_*t*_) with various climatic variables when setting the conversion rate to 0.5. **Fig. S25.** Exposure-response curves of the associations of daily instantaneous effective reproductive number (R_*t*_) with various climatic variables when setting the conversion rate to 0.05. **Fig. S26.** Exposure-response curves of the associations of daily instantaneous effective reproductive number (R_*t*_) with various climatic variables when setting the conversion rate to 0.005. **Fig. S27.** Exposure-response curves of the associations of daily instantaneous effective reproductive number (R_*t*_) with various climatic variables when setting the mean and standard deviation of serial interval for influenza to 3.3 and 1.7, respectively. **Fig. S28.** Exposure-response curves of the associations of daily instantaneous effective reproductive number (R_*t*_) with various climatic variables when setting the mean and standard deviation of serial interval for influenza to 2.6 and 1.5, respectively. **Fig. S29.** Exposure-response curves of the associations of daily instantaneous effective reproductive number (R_*t*_) with various climatic variables when considering data from maximum 8 weeks either side of each epidemic peak. **Fig. S30.** Exposure-response curves of the associations of daily instantaneous effective reproductive number (R_*t*_) with various climatic variables when considering data from maximum 10 weeks either side of each epidemic peak. **Table S4.** Comparisons of studies which assessed the association between meteorological factors and influenza transmissibility.

## Data Availability

The datasets used and/or analyzed during the current study are available from the corresponding authors on reasonable request.

## Abbreviations

CDC: Guangzhou Center for Disease Control and Prevention
DTR: Diurnal temperature range
ERA5-Land: The fifth generation of European Reanalysis Land
HTV: Hourly temperature variability
ILI: Influenza-like illness
PLADs: Provincial-level administrative divisions
*R*_*t*_: Effective reproductive number
