# Supplementary material for: Effects of meteorological factors on influenza transmissibility by virus type/subtype
Source: BMC Public Health. 2024 Feb 16;24:494. doi: 10.1186/s12889-024-17961-9 (PMC10870479; doi:10.1186/s12889-024-17961-9)
Supplement: Supplementary file 1 — Additional file 1. Definition of influenza epidemics. Models. Fig. S1. Estimates of daily instantaneous effective reproductive number (Rt) for each influenza epidemic used in the gamma regression analysis. Fig. S2. Estimates of daily instantaneous effective reproductive number (Rt) for each influenza A(H1N1)pdm09 epidemic used in the gamma regression analysis. Fig. S3. Estimates of daily instantaneous effective reproductive number (Rt) for each influenza A(H3N2) epidemic used in the gamma regression analysis. Fig. S4. Estimates of daily instantaneous effective reproductive number (Rt) for each influenza B epidemic used in the gamma regression analysis. Table S1. Results of Wald tests of the statistical significance of each meteorological factor. Table S2. Summary statistics of the estimates of daily instantaneous effective reproductive number when using different conversion rates to calculate influenza incidence. Fig. S5. Estimates of daily instantaneous effective reproductive number (Rt) for each influenza epidemic when setting the conversion rate to 0.5. Fig. S6. Estimates of daily instantaneous effective reproductive reproduction number (Rt) for each influenza A(H1N1)pdm09 epidemic when setting the conversion rate to 0.5. Fig. S7. Estimates of daily instantaneous effective reproductive number (Rt) for each influenza A(H3N2) epidemic when setting the conversion rate to 0.5. Fig. S8. Estimates of daily instantaneous effective reproductive number (Rt) for each influenza B epidemic when setting the conversion rate to 0.5. Fig. S9. Estimates of daily instantaneous effective reproductive number (Rt) for each influenza epidemic when setting the conversion rate to 0.05. Fig. S10. Estimates of daily instantaneous effective reproductive number (Rt) for each influenza A(H1N1)pdm09 epidemic when setting the conversion rate to 0.05. Fig. S11. Estimates of daily instantaneous effective reproductive number (Rt) for each influenza A(H3N2) epidemic when setting the conversion rat [file 12889_2024_17961_MOESM1_ESM.docx]

# Supplementary Materials

**Effects of meteorological factors on influenza transmissibility by virus type/subtype**

Ze-Lin Yan^1#^, Wen-Hui Liu^1,2#^, Yu-Xiang Long^1^, Bo-Wen Ming^1^, Zhou Yang^1^, Peng-Zhe Qin^2*^, Chun-Quan Ou^1*^, Li Li^1*^

^1^ State Key Laboratory of Organ Failure Research, Department of Biostatistics, Guangdong Provincial Key Laboratory of Tropical Disease Research, School of Public Health, Southern Medical University, Guangzhou, Guangdong, China

^2^ Guangzhou Center for Disease Control and Prevention, Guangzhou, Guangdong, China

^#^ Contributed equally as co-first authors

^*^ Contributed equally as co-corresponding authors:

Li Li (Email: lylygdsg@163.com)

Chun-Quan Ou (Email: [ouchunquan@hotmail.com](mailto:ouchunquan@hotmail.com))

Peng-Zhe Qin (Email: [petgyy@gmail.com](mailto:petgyy@gmail.com))

## Additional file 1: Definition of influenza epidemics

We defined influenza epidemics as periods of at least five consecutive weeks during which an epidemic threshold (i.e., the median of all non-zero weekly ILI+ during the study period [1]) was exceeded, and the largest two ILI+ exceeded the 70^th^ percentile of all non-zero weekly ILI+. The influenza epidemic ended when ILI+ was less than the epidemic threshold for at least two consecutive weeks. Influenza epidemics were identified for each influenza type/subtype separately, and the epidemics of different influenza types/subtypes were combined to determine the epidemics of all influenza. Two epidemics were combined into one epidemic if there were less than three weeks between the two epidemics.

## Additional file 2: Models

A gamma regression with a log link combined with a distributed lag non-linear model was used to assess the potential non-linear effects of meteorological factors on influenza transmissibility [2]. Our analysis was restricted to the data from maximum of nine weeks either side of the peak of influenza epidemic. The model for assessing the association between temperature and influenza transmissibility is expressed as follows:

$$\log\left[ E\left( R_{t} \right) \right]=\alpha_{0}+\alpha_{1}{Epi}_{t}+\alpha_{2}{Holiday}_{t}+ns({Cum}_{t},df=3)$$

$$+\alpha_{3}{Temp}_{t,l}+\alpha_{4}{RH}_{t,l} Model 1$$

where $E\left( R_{t} \right)$ is the expected median instantaneous effective reproductive number at time $t$ for all influenza or for each influenza type/subtype, ${Epi}_{t}$ represents the influenza epidemic at time $t$, ${Holiday}_{t}$ is an indicator variable (1 = public holidays, weekends, winter holidays, and summer holidays; 0 = other days), and ${Cum}_{t}$ means the cumulative number of influenza infections up to the time $t$-1 of an influenza epidemic. We applied a natural cubic spline with three degrees of freedom (*df*s) to ${Cum}_{t}$. ${Temp}_{t,l}$ and ${RH}_{t,l}$ are two-dimensional cross-basis matrices of meteorological factors (i.e., daily mean temperature and relative humidity) and lag, respectively, which were fitted using natural cubic splines with three *df*s. The maximum lag $l$ was set to 14 days [1, 3, 4].

The basic model for examining the association between HTV and influenza transmissibility is as follows:

$$\log\left[ E\left( R_{t} \right) \right]=\beta_{0}+\beta_{1}{Epi}_{t}+\beta_{2}{Holiday}_{t}+ns({Cum}_{t},df=3)$$

$$+\beta_{3}{Temp}_{t,l}+ns\left( {HTV}_{t},df=3 \right) Model 2$$

where ${HTV}_{t}$ is the hourly temperature variability over 0–14 days at time $t$. A natural cubic spline with three *df*s was applied to the HTV. In the analysis of the association between the HTV and *R_t_*, absolute or relative humidity was added to Model 2 to obtain Models 3 and 4. The models with the smallest Akaike information criterion (AIC) were selected. Model 4 was fitted to assess the association between relative humidity and influenza transmissibility.

Temperature and absolute humidity were commonly highly correlated; therefore, these two variables were not included in the same model when investigating the effects of each of these variables on influenza transmissibility. The following model is constructed to evaluate the association between absolute humidity and influenza transmissibility:

$$\log\left[ E\left( R_{t} \right) \right]=\gamma_{0}+\gamma_{1}{Epi}_{t}+\gamma_{2}{Holiday}_{t}+ns({Cum}_{t},df=3)$$

$$+\gamma_{3}{AH}_{t,l}+ns\left( {HTV}_{t},df=3 \right) Model 5$$

where ${AH}_{t,l}$ is the two-dimensional cross-basis matrix of absolute humidity and lag. The same maximum lag and functions as the basis of variables and lags for temperature and relative humidity were applied to absolute humidity. The partial autocorrelation function of the residuals of the aforementioned models suggests a high autocorrelation at lags of one and two days. Thus, we also included the autoregression terms of lag one and lag two days in the models [5].


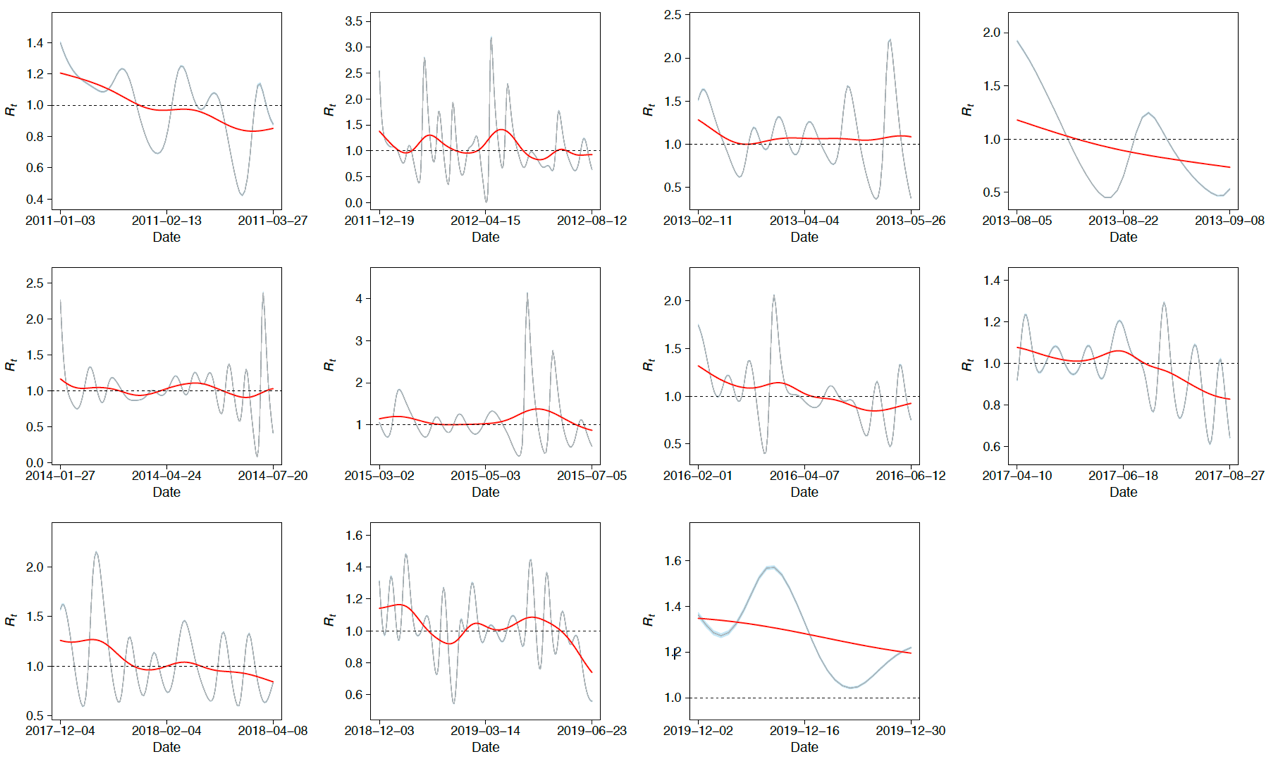


**Additional file 3: Figure S1.** Estimates of daily instantaneous effective reproductive number (*R_t_*) for each influenza epidemic used in the gamma regression analysis. Grey lines and light blue areas represent the point estimates of *R_t_* and the corresponding 95% credible intervals, respectively. Red lines indicate the smoothed *R_t_* estimates.


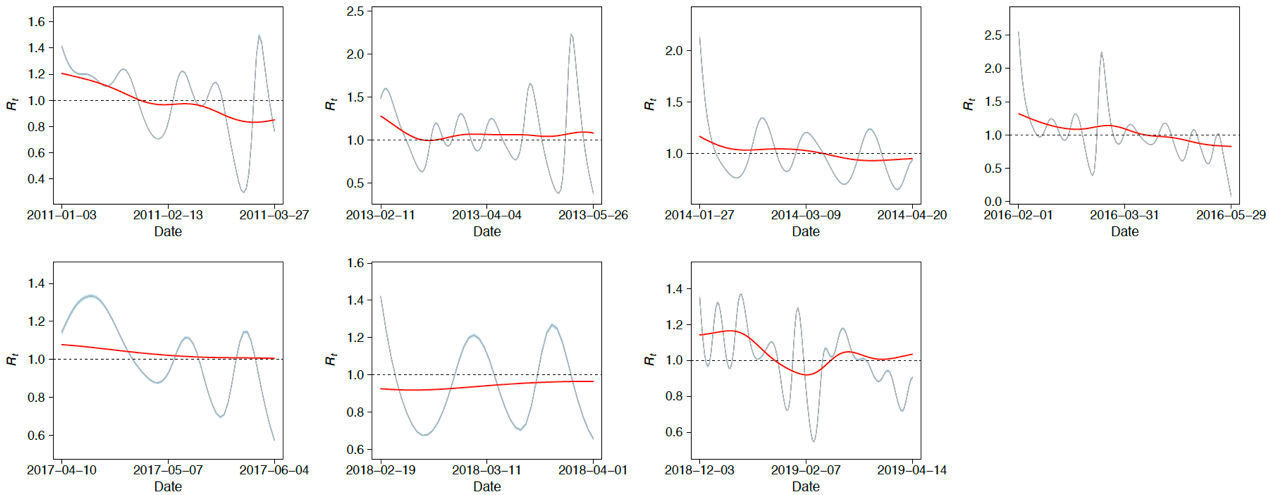


**Additional file 4: Figure S2.** Estimates of daily instantaneous effective reproductive number (*R_t_*) for each influenza A(H1N1)pdm09 epidemic used in the gamma regression analysis. Grey lines and light blue areas are the point estimates of *R_t_* and the corresponding 95% credible intervals, respectively. Red lines indicate the smoothed *R_t_* estimates.


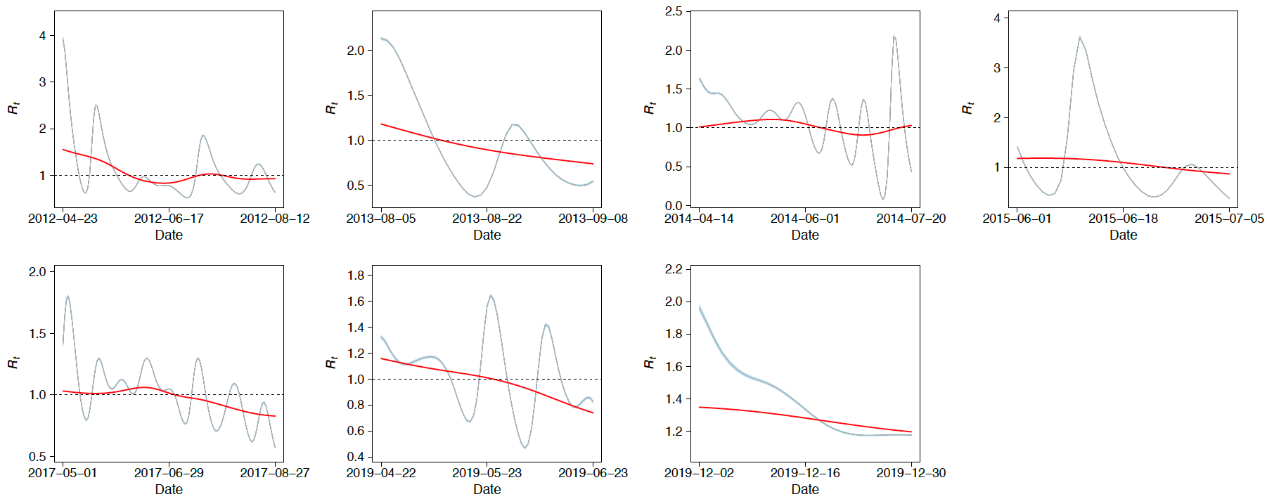


**Additional file 5: Figure S3.** Estimates of daily instantaneous effective reproductive number (*R_t_*) for each influenza A(H3N2) epidemic used in the gamma regression analysis. Grey lines and light blue areas represent the point estimates of *R_t_* and the corresponding 95% credible intervals, respectively. Red lines indicate the smoothed *R_t_* estimates.


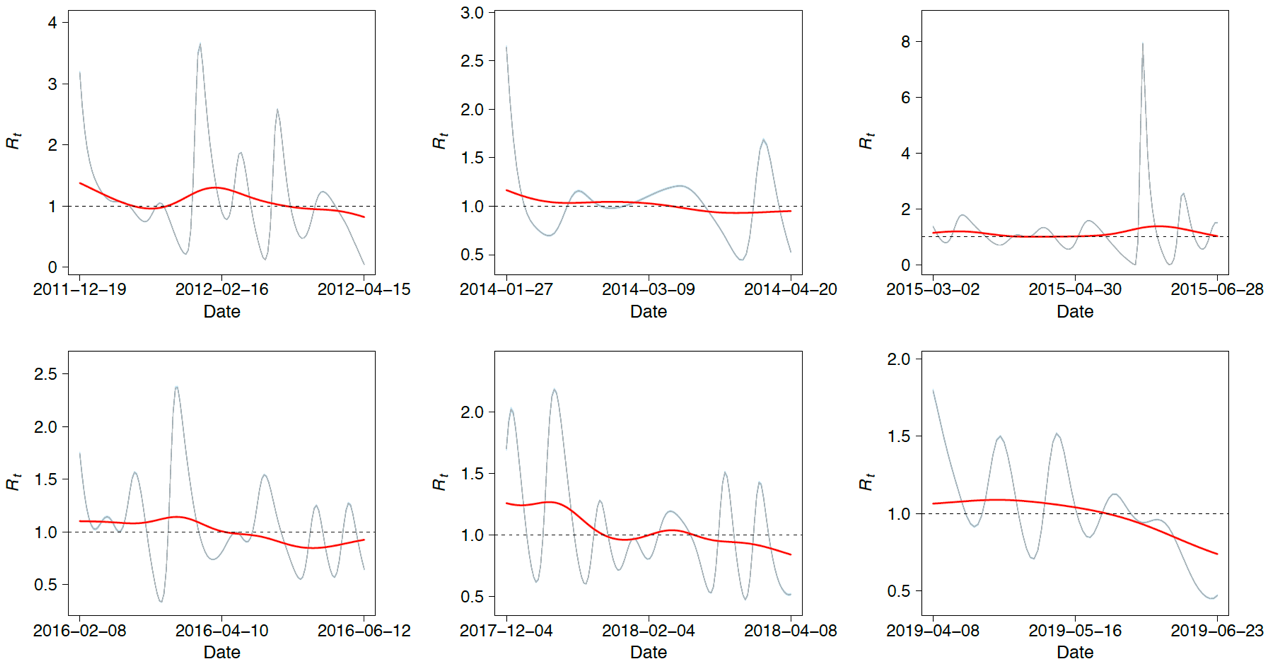


**Additional file 6: Figure S4.** Estimates of daily instantaneous effective reproductive number (*R_t_*) for each influenza B epidemic used in the gamma regression analysis. Grey lines and light blue areas are the point estimates of *R_t_* and the corresponding 95% credible intervals, respectively. Red lines indicate the smoothed *R_t_* estimates.

**Additional file 7: Table S1.** Results of Wald tests of the statistical significance of each meteorological factor

| Variables | Influenza epidemics | *F* | *P* |
| --- | --- | --- | --- |
| Temperature | All influenza | 177.519 | <0.001 |
|  | A(H1N1)pdm09 | 169.552 | <0.001 |
|  | A(H3N2) | 58.945 | <0.001 |
|  | B | 114.777 | <0.001 |
| Hourly temperature variability | All influenza | 17.841 | <0.001 |
|  | A(H1N1)pdm09 | 64.151 | <0.001 |
|  | A(H3N2) | 49.175 | <0.001 |
|  | B | 37.713 | <0.001 |
| Absolute humidity | All influenza | 150.879 | <0.001 |
|  | A(H1N1)pdm09 | 169.684 | <0.001 |
|  | A(H3N2) | 87.404 | <0.001 |
|  | B | 90.070 | <0.001 |
| Relative humidity | All influenza | 196.882 | <0.001 |
|  | A(H1N1)pdm09 | 147.501 | <0.001 |
|  | A(H3N2) | 46.145 | <0.001 |
|  | B | 128.613 | <0.001 |

**Additional file 8: Table S2.** Summary statistics of the estimates of daily instantaneous effective reproductive number when using different conversion rates to calculate influenza incidence

| Conversion  rate | Influenza epidemics | Mean | *SD* | Minimum | *P*_25_ | Median | *P*_75_ | Maximum |
| --- | --- | --- | --- | --- | --- | --- | --- | --- |
| 0.5 | All influenza | 1.044 | 0.379 | 0.000 | 0.836 | 1.007 | 1.185 | 4.129 |
|  | A(H1N1)pdm09 | 1.017 | 0.287 | 0.080 | 0.855 | 1.009 | 1.171 | 2.543 |
|  | A(H3N3) | 1.086 | 0.477 | 0.072 | 0.778 | 1.049 | 1.251 | 3.934 |
|  | B | 1.087 | 0.610 | 0.000 | 0.769 | 1.005 | 1.246 | 7.920 |
| 0.05 | All influenza | 1.044 | 0.380 | 0.000 | 0.836 | 1.007 | 1.185 | 4.131 |
|  | A(H1N1)pdm09 | 1.017 | 0.287 | 0.081 | 0.855 | 1.009 | 1.171 | 2.542 |
|  | A(H3N3) | 1.086 | 0.477 | 0.072 | 0.777 | 1.049 | 1.251 | 3.931 |
|  | B | 1.087 | 0.609 | 0.000 | 0.769 | 1.005 | 1.246 | 7.916 |
| 0.005 | All influenza | 1.044 | 0.379 | 0.003 | 0.836 | 1.008 | 1.185 | 4.137 |
|  | A(H1N1)pdm09 | 1.017 | 0.287 | 0.087 | 0.856 | 1.009 | 1.171 | 2.539 |
|  | A(H3N3) | 1.087 | 0.477 | 0.075 | 0.778 | 1.050 | 1.251 | 3.919 |
|  | B | 1.087 | 0.608 | 0.001 | 0.770 | 1.007 | 1.249 | 7.867 |

Abbreviations: *SD*, standard deviation; *P*_25_, the 25^th^ percentile; *P*_75_, the 75^th^ percentile.


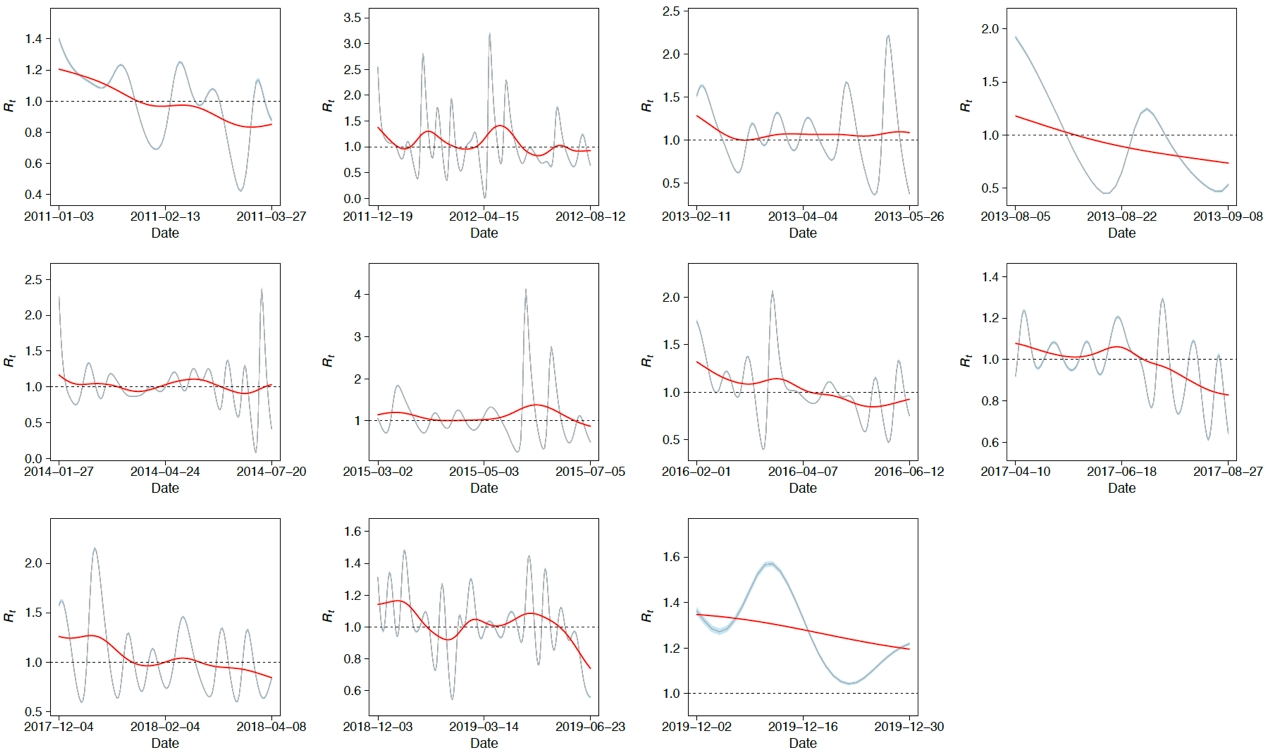


**Additional file 9: Figure S5.** Estimates of daily instantaneous effective reproductive number (*R_t_*) for each influenza epidemic when setting the conversion rate to 0.5. Grey lines and light blue areas are the point estimates of *R_t_* and the corresponding 95% credible intervals, respectively. Red lines indicate the smoothed *R_t_* estimates.


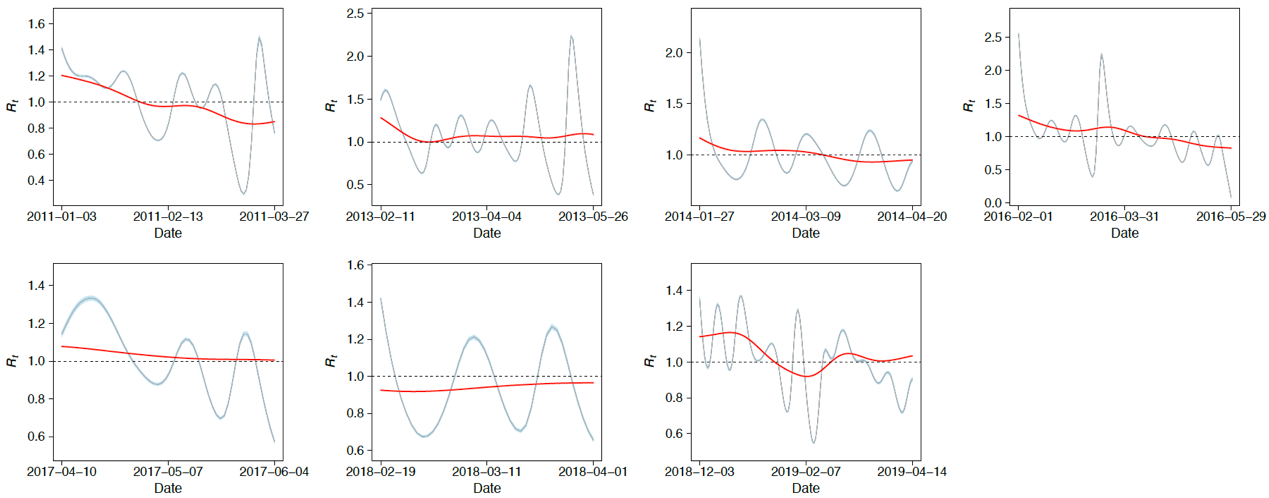


**Additional file 10: Figure S6.** Estimates of daily instantaneous effective reproductive number (*R_t_*) for each influenza A(H1N1)pdm09 epidemic when setting the conversion rate to 0.5. Grey lines and light blue areas are the point estimates of *R_t_* and the corresponding 95% credible intervals, respectively. Red lines indicate the smoothed *R_t_* estimates.


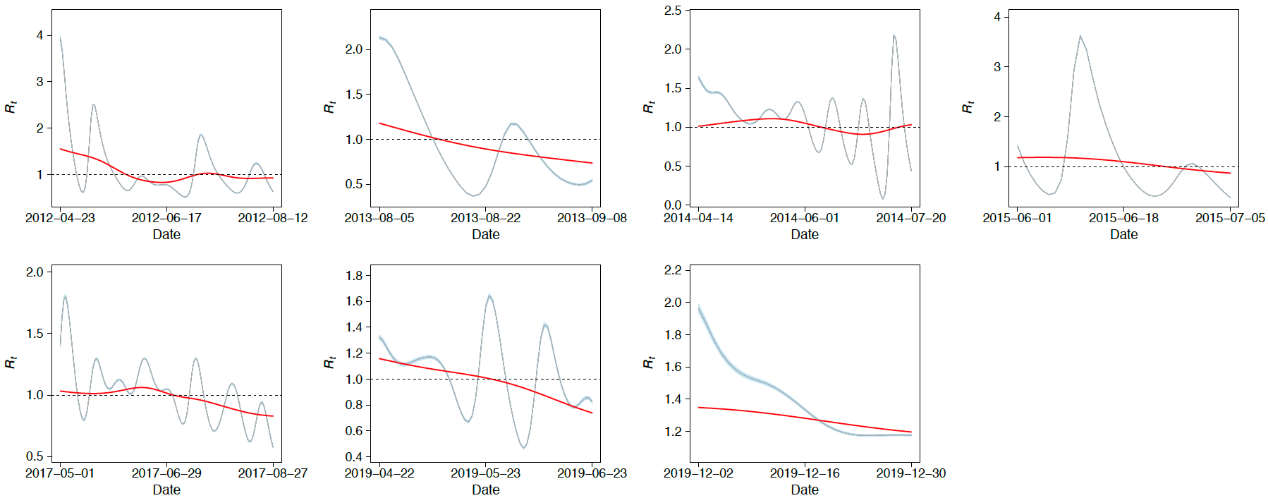


**Additional file 11: Figure S7.** Estimates of daily instantaneous effective reproductive number (*R_t_*) for each influenza A(H3N2) epidemic when setting the conversion rate to 0.5. Grey lines and light blue areas are the point estimates of *R_t_* and the corresponding 95% credible intervals, respectively. Red lines indicate the smoothed *R_t_* estimates.


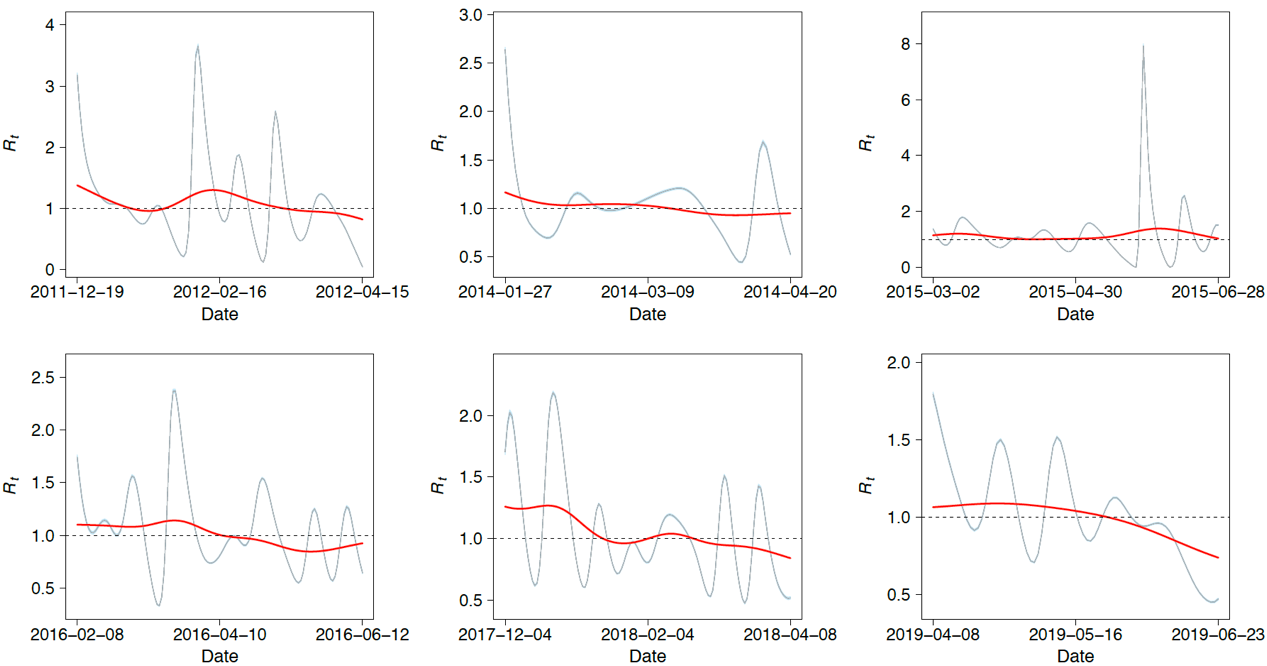


**Additional file 12: Figure S8.** Estimates of daily instantaneous effective reproductive number (*R_t_*) for each influenza B epidemic when setting the conversion rate to 0.5. Grey lines and light blue areas are the point estimates of *R_t_* and the corresponding 95% credible intervals, respectively. Red lines indicate the smoothed *R_t_* estimates.

**Additional file 13: Figure S9.** Estimates of daily instantaneous effective reproductive number (*R_t_*) for each influenza epidemic when setting the conversion rate to 0.05. Grey lines and light blue areas are the point estimates of *R_t_* and the corresponding 95% credible intervals, respectively. Red lines indicate the smoothed *R_t_* estimates.

**Additional file 14: Figure S10.** Estimates of daily instantaneous effective reproductive number (*R_t_*) for each influenza A(H1N1)pdm09 epidemic when setting the conversion rate to 0.05. Grey lines and light blue areas are the point estimates of *R_t_* and the corresponding 95% credible intervals, respectively. Red lines indicate the smoothed *R_t_* estimates.

**Additional file 15: Figure S11.** Estimates of daily instantaneous effective reproductive number (*R_t_*) for each influenza A(H3N2) epidemic when setting the conversion rate to 0.05. Grey lines and light blue areas are the point estimates of *R_t_* and the corresponding 95% credible intervals, respectively. Red lines indicate the smoothed *R_t_* estimates.

**Additional file 16: Figure S12.** Estimates of daily instantaneous effective reproductive number (*R_t_*) for each influenza B epidemic when setting the conversion rate to 0.05. Grey lines and light blue areas are the point estimates of *R_t_* and the corresponding 95% credible intervals, respectively. Red lines indicate the smoothed *R_t_* estimates.

**Additional file 17: Figure S13.** Estimates of daily instantaneous effective reproductive number (*R_t_*) for each influenza epidemic when setting the conversion rate to 0.005. Grey lines and light blue areas are the point estimates of *R_t_* and the corresponding 95% credible intervals, respectively. Red lines indicate the smoothed *R_t_* estimates.

**Additional file 18: Figure S14.** Estimates of daily instantaneous effective reproductive number (*R_t_*) for each influenza A(H1N1)pdm09 epidemic when setting the conversion rate to 0.005. Grey lines and light blue areas are the point estimates of *R_t_* and the corresponding 95% credible intervals, respectively. Red lines indicate the smoothed *R_t_* estimates.

**Additional file 19: Figure S15.** Estimates of daily instantaneous effective reproductive number (*R_t_*) for each influenza A(H3N2) epidemic when setting the conversion rate to 0.005. Grey lines and light blue areas are the point estimates of *R_t_* and the corresponding 95% credible intervals, respectively. Red lines indicate the smoothed *R_t_* estimates.

**Additional file 20: Figure S16.** Estimates of daily instantaneous effective reproductive number (*R_t_*) for each influenza B epidemic when setting the conversion rate to 0.005. Grey lines and light blue areas are the point estimates of *R_t_* and the corresponding 95% credible intervals, respectively. Red lines indicate the smoothed *R_t_* estimates.

**Additional file 21: Table S3.** Summary statistics of the estimates of daily instantaneous effective reproductive number when assuming different means and *SD*s of the serial interval

| Mean ± *SD*  of the serial interval | Influenza epidemics | Mean | *SD* | Minimum | *P*_25_ | Median | *P*_75_ | Maximum |
| --- | --- | --- | --- | --- | --- | --- | --- | --- |
| 3.3 ± 1.7 | All influenza | 1.044 | 0.379 | 0.000 | 0.836 | 1.007 | 1.185 | 4.129 |
|  | A(H1N1)pdm09 | 1.018 | 0.297 | 0.076 | 0.846 | 1.012 | 1.177 | 2.674 |
|  | A(H3N3) | 1.082 | 0.465 | 0.076 | 0.785 | 1.047 | 1.236 | 3.992 |
|  | B | 1.076 | 0.586 | 0.000 | 0.787 | 1.007 | 1.226 | 8.019 |
| 2.6 ± 1.5 | All influenza | 1.025 | 0.301 | 0.000 | 0.864 | 1.005 | 1.144 | 3.236 |
|  | A(H1N1)pdm09 | 1.005 | 0.236 | 0.097 | 0.870 | 1.007 | 1.137 | 2.080 |
|  | A(H3N3) | 1.048 | 0.355 | 0.103 | 0.822 | 1.037 | 1.191 | 2.983 |
|  | B | 1.045 | 0.474 | 0.000 | 0.822 | 1.002 | 1.172 | 7.203 |

Abbreviations: *SD*, standard deviation; *P*_25_, the 25^th^ percentile; *P*_75_, the 75^th^ percentile.

**Additional file 22: Figure S17.** Estimates of daily instantaneous effective reproductive number (*R_t_*) for each influenza A(H1N1)pdm09 epidemic when assuming the mean and standard deviation of serial interval were 3.3 and 1.7, respectively. Grey lines and light blue areas are the point estimates of *R_t_* and the corresponding 95% credible intervals, respectively. Red lines indicate the smoothed *R_t_* estimates.

**Additional file 23: Figure S18.** Estimates of daily instantaneous effective reproductive number (*R_t_*) for each influenza A(H3N2) epidemic when assuming the mean and standard deviation of serial interval were 3.3 and 1.7, respectively. Grey lines and light blue areas are the point estimates of *R_t_* and the corresponding 95% credible intervals, respectively. Red lines indicate the smoothed *R_t_* estimates.

**Additional file 24: Figure S19.** Estimates of daily instantaneous effective reproductive number (*R_t_*) for each influenza B epidemic when assuming the mean and standard deviation of serial interval were 3.3 and 1.7, respectively. Grey lines and light blue areas are the point estimates of *R_t_* and the corresponding 95% credible intervals, respectively. Red lines indicate the smoothed *R_t_* estimates.

**Additional file 25: Figure S20.** Estimates of daily instantaneous effective reproductive number (*R_t_*) for each influenza epidemic when assuming the mean and standard deviation of serial interval were 2.6 and 1.5, respectively. Grey lines and light blue areas are the point estimates of *R_t_* and the corresponding 95% credible intervals, respectively. Red lines indicate the smoothed *R_t_* estimates.

**Additional file 26: Figure S21.** Estimates of daily instantaneous effective reproductive number (*R_t_*) for each influenza A(H1N1)pdm09 epidemic when assuming the mean and standard deviation of serial interval were 2.6 and 1.5, respectively. Grey lines and light blue areas are the point estimates of *R_t_* and the corresponding 95% credible intervals, respectively. Red lines indicate the smoothed *R_t_* estimates.

**Additional file 27: Figure S22.** Estimates of daily instantaneous effective reproductive number (*R_t_*) for each influenza A(H3N2) epidemic when assuming the mean and standard deviation of serial interval were 2.6 and 1.5, respectively. Grey lines and light blue areas are the point estimates of *R_t_* and the corresponding 95% credible intervals, respectively. Red lines indicate the smoothed *R_t_* estimates.

**Additional file 28: Figure S23.** Estimates of daily instantaneous effective reproductive number (*R_t_*) for each influenza B epidemic when assuming the mean and standard deviation of serial interval were 2.6 and 1.5, respectively. Grey lines and light blue areas are the point estimates of *R_t_* and the corresponding 95% credible intervals, respectively. Red lines indicate the smoothed *R_t_* estimates.


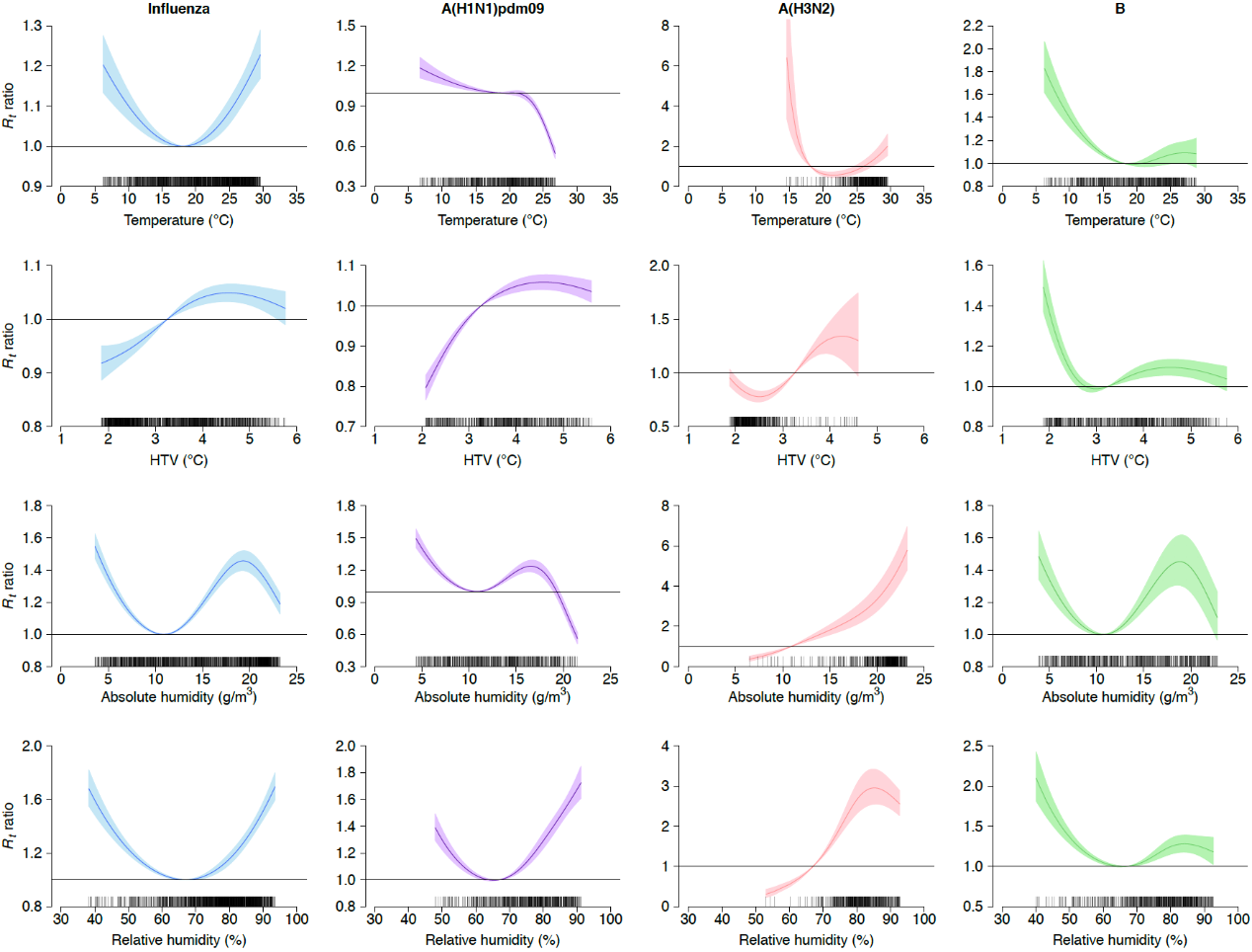


**Additional file 29: Figure S24.** Exposure-response curves of the associations of daily instantaneous effective reproductive number (*R_t_*) with various climatic variables when setting the conversion rate to 0.5. Curves and colored areas represent the point estimates of *R_t_* ratios and the corresponding confidence intervals, respectively. The ticks along the x-axis are observed meteorological data. Horizontal lines indicating *R_t_* ratio = 1 were also plotted. The *R_t_* ratios are the ratio of predicted *R_t_* with respect to reference values for the meteorological factors of mean temperature, hourly temperature variability (HTV), absolute humidity, and relative humidity set to 18.12°C, 3.25°C, 10.88g/m^3^, and 66.99%, respectively. We depicted the associations excluding the ten lowest and the ten largest values of meteorological factors, avoiding the potentially unrobust estimates due to small sample size.


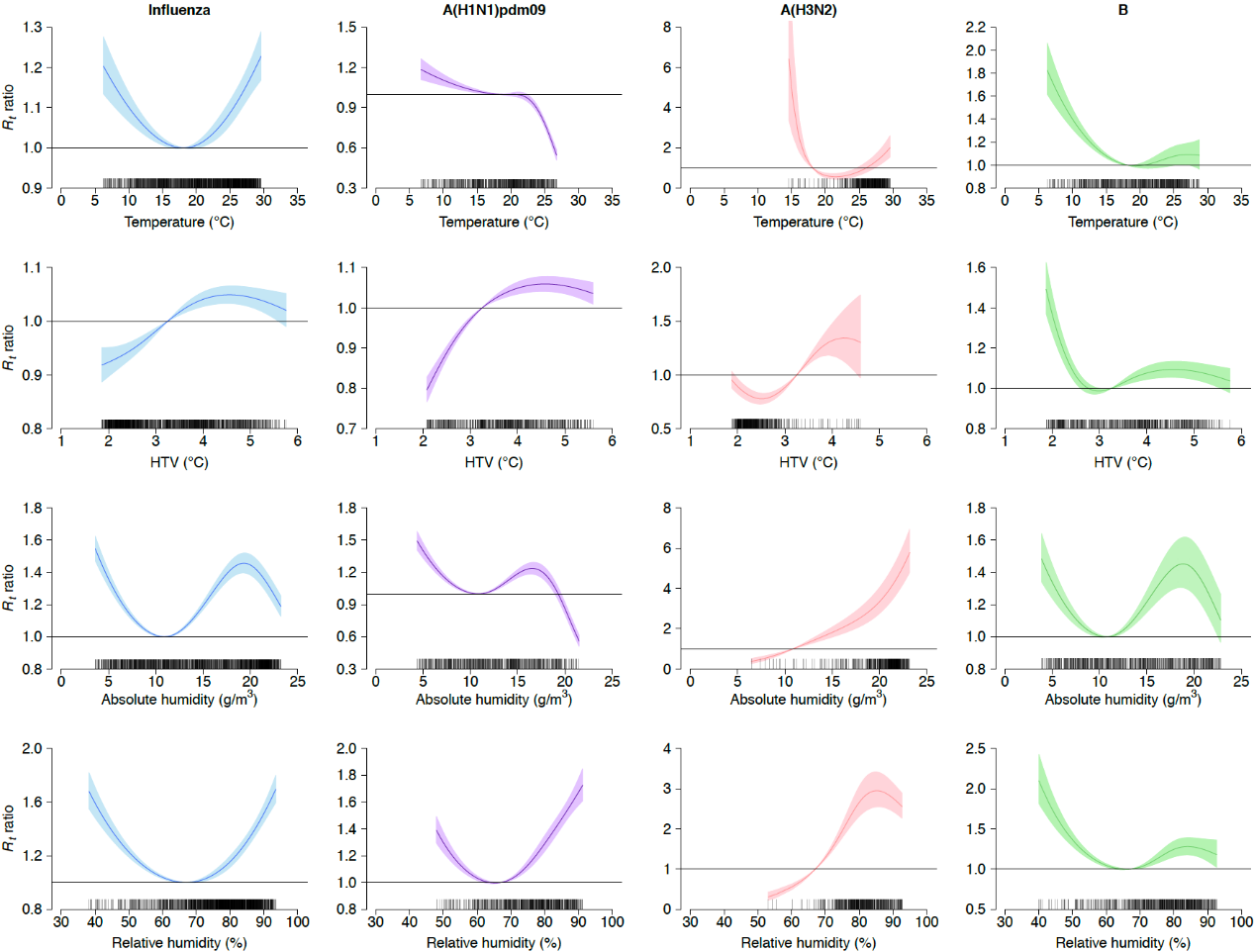


**Additional file 30: Figure S25.** Exposure-response curves of the associations of daily instantaneous effective reproductive number (*R_t_*) with various climatic variables when setting the conversion rate to 0.05. Curves and colored areas represent the point estimates of *R_t_* ratios and the corresponding confidence intervals, respectively. The ticks along the x-axis are observed meteorological data. Horizontal lines indicating *R_t_* ratio = 1 were also plotted. The *R_t_* ratios are the ratio of predicted *R_t_* with respect to reference values for the meteorological factors of mean temperature, hourly temperature variability (HTV), absolute humidity, and relative humidity set to 18.12°C, 3.25°C, 10.88g/m^3^, and 66.99%, respectively. We depicted the associations excluding the ten lowest and the ten largest values of meteorological factors, avoiding the potentially unrobust estimates due to small sample size.


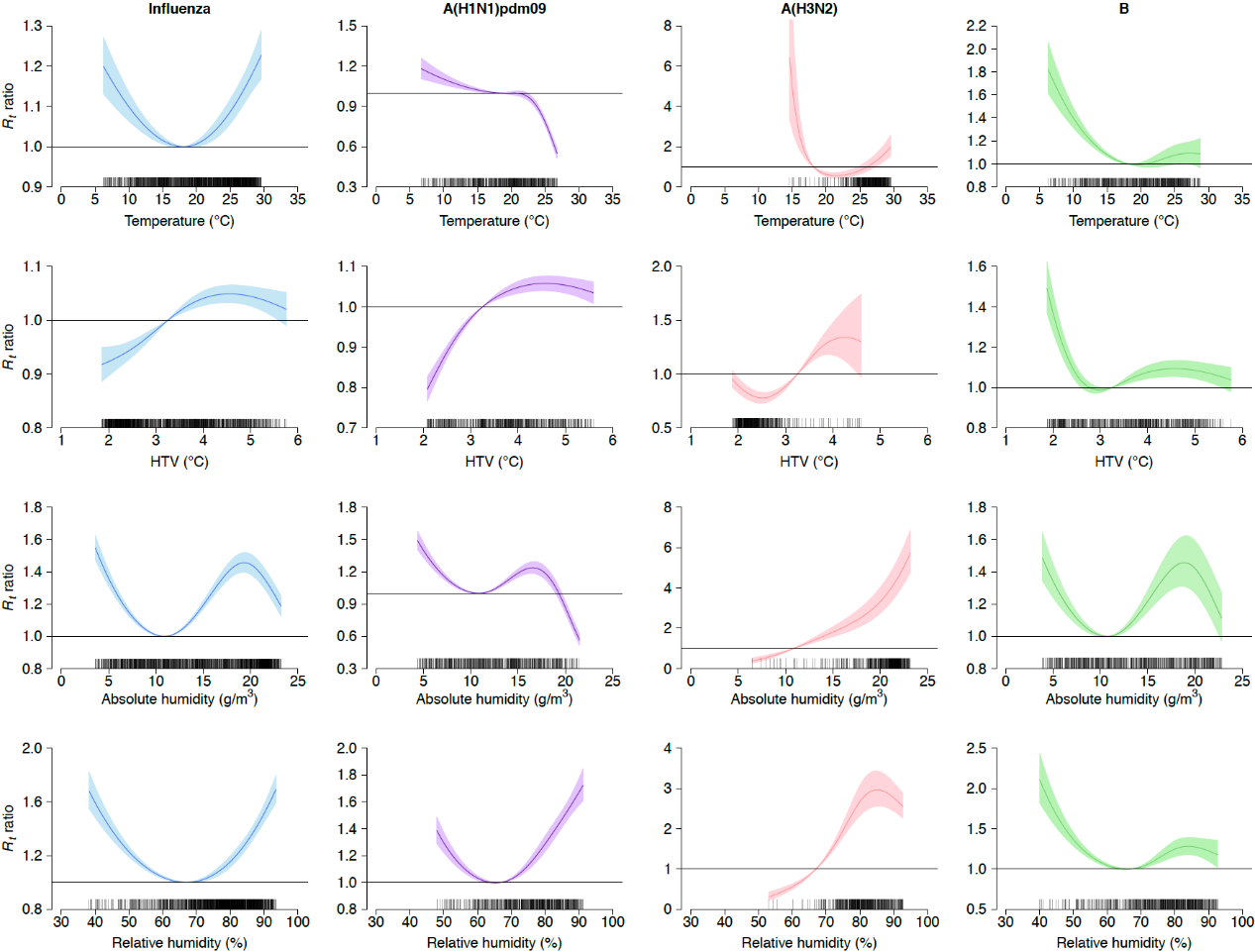


**Additional file 31: Figure S26.** Exposure-response curves of the associations of daily instantaneous effective reproductive number (*R_t_*) with various climatic variables when setting the conversion rate to 0.005. Curves and colored areas represent the point estimates of *R_t_* ratios and the corresponding confidence intervals, respectively. The ticks along the x-axis are observed meteorological data. Horizontal lines indicating *R_t_* ratio = 1 were also plotted. The *R_t_* ratios are the ratio of predicted *R_t_* with respect to reference values for the meteorological factors of mean temperature, hourly temperature variability (HTV), absolute humidity, and relative humidity set to 18.12°C, 3.25°C, 10.88g/m^3^, and 66.99%, respectively. We depicted the associations excluding the ten lowest and the ten largest values of meteorological factors, avoiding the potentially unrobust estimates due to small sample size.


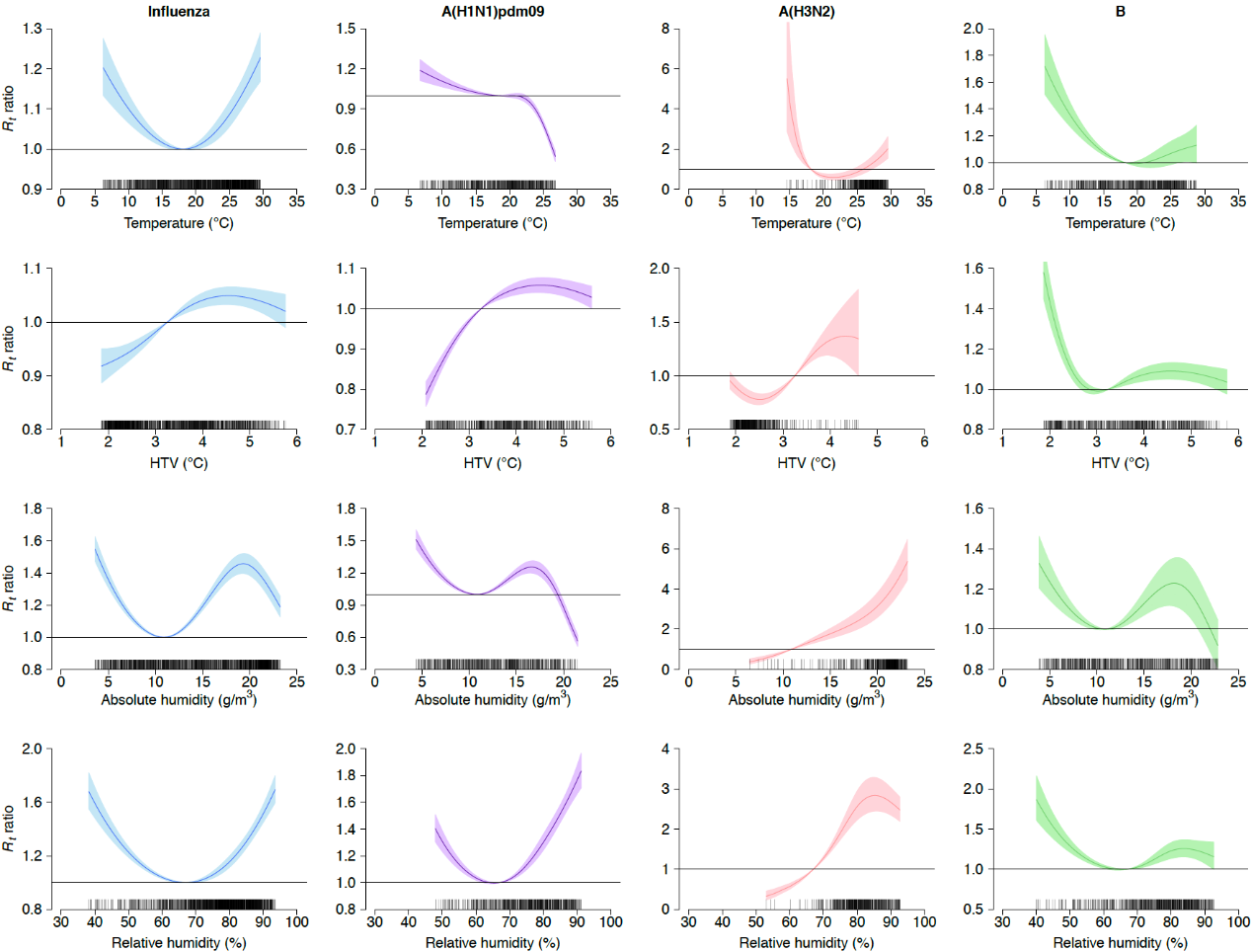


**Additional file 32: Figure S27.** Exposure-response curves of the associations of daily instantaneous effective reproductive number (*R_t_*) with various climatic variables when setting the mean and standard deviation of serial interval for influenza to 3.3 and 1.7, respectively. Curves and colored areas represent the point estimates of *R_t_* ratios and the corresponding confidence intervals, respectively. The ticks along the x-axis are observed meteorological data. Horizontal lines indicating *R_t_* ratio = 1 were also plotted. The *R_t_* ratios are the ratio of predicted *R_t_* with respect to reference values for the meteorological factors of mean temperature, hourly temperature variability (HTV), absolute humidity, and relative humidity set to 18.12°C, 3.25°C, 10.88g/m^3^, and 66.99%, respectively. We depicted the associations excluding the ten lowest and the ten largest values of meteorological factors, avoiding the potentially unrobust estimates due to small sample size.

**
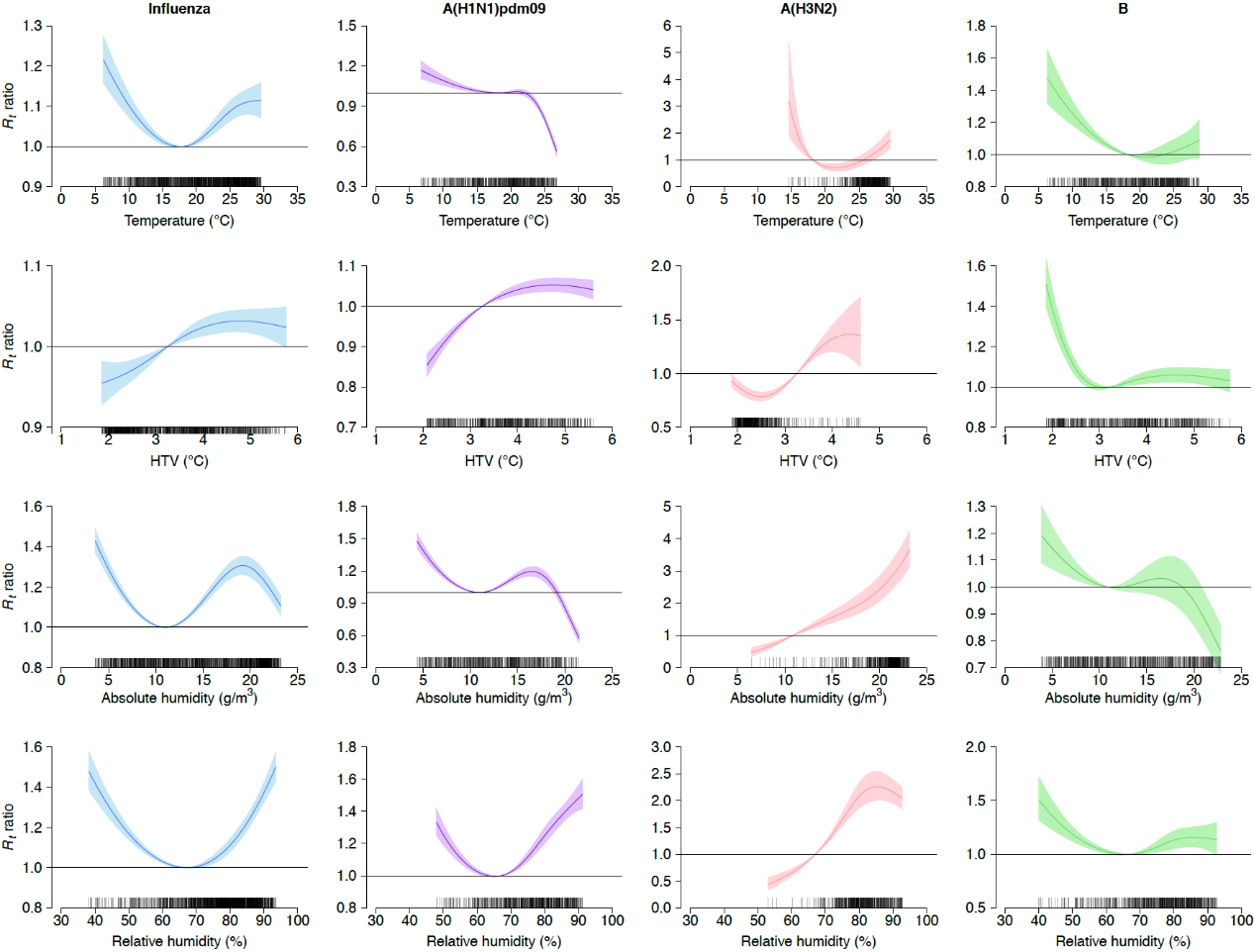
**

**Additional file 33: Figure S28.** Exposure-response curves of the associations of daily instantaneous effective reproductive number (*R_t_*) with various climatic variables when setting the mean and standard deviation of serial interval for influenza to 2.6 and 1.5, respectively. Curves and colored areas represent the point estimates of *R_t_* ratios and the corresponding confidence intervals, respectively. The ticks along the x-axis are observed meteorological data. Horizontal lines indicating *R_t_* ratio = 1 were also plotted. The *R_t_* ratios are the ratio of predicted *R_t_* with respect to reference values for the meteorological factors of mean temperature, hourly temperature variability (HTV), absolute humidity, and relative humidity set to 18.12°C, 3.25°C, 10.88g/m^3^, and 66.99%, respectively. We depicted the associations excluding the ten lowest and the ten largest values of meteorological factors, avoiding the potentially unrobust estimates due to small sample size.


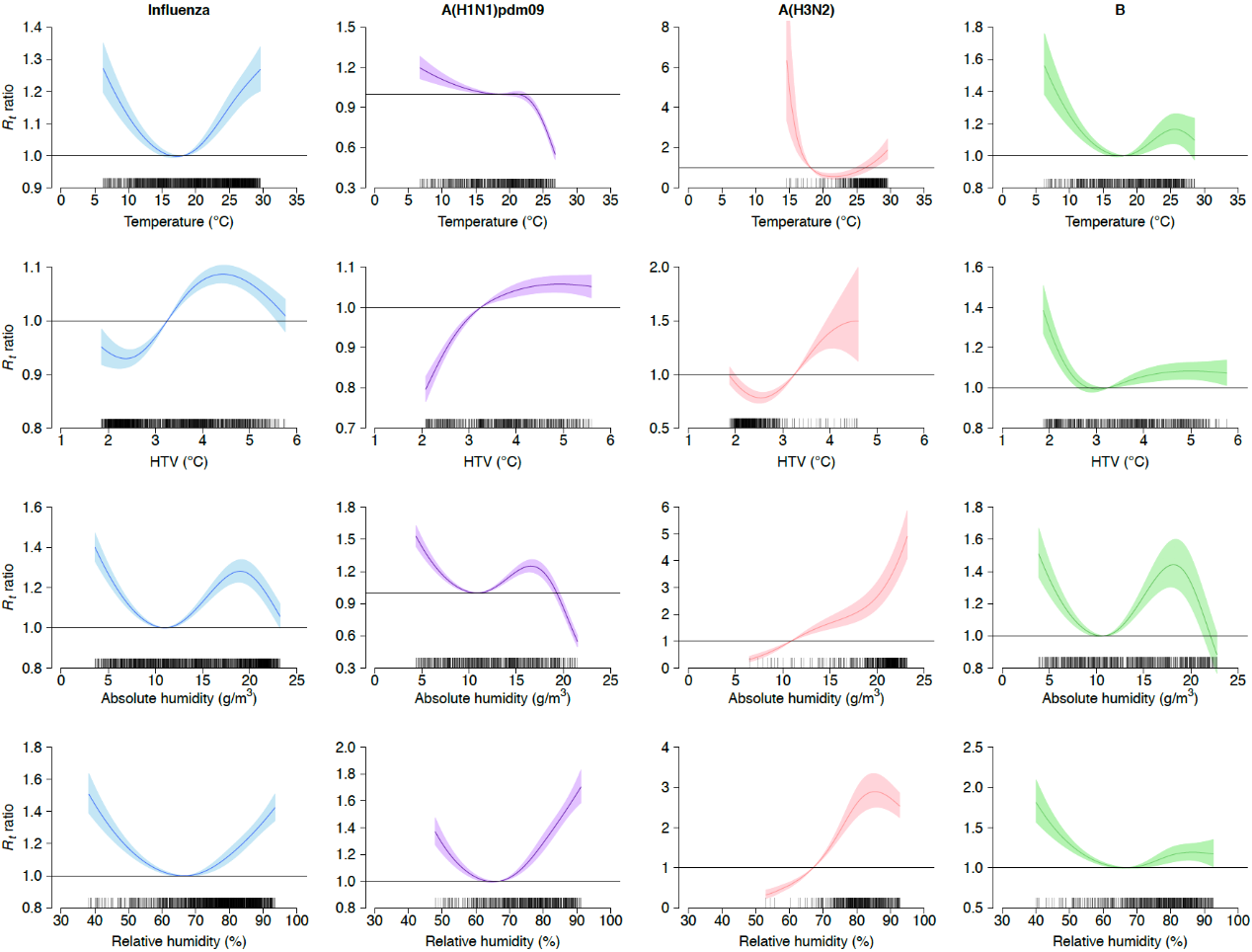
**Additional file 34: Figure S29.** Exposure-response curves of the associations of daily instantaneous effective reproductive number (*R_t_*) with various climatic variables when considering data from maximum eight weeks either side of each epidemic peak. Curves and colored areas represent the point estimates of *R_t_* ratios and the corresponding confidence intervals, respectively. The ticks along the x-axis are observed meteorological data. Horizontal lines indicating *R_t_* ratio = 1 were also plotted. The *R_t_* ratios are the ratio of predicted *R_t_* with respect to reference values for the meteorological factors of mean temperature, hourly temperature variability (HTV), absolute humidity, and relative humidity set to 18.12°C, 3.25°C, 10.88g/m^3^, and 66.99%, respectively. We depicted the associations excluding the ten lowest and the ten largest values of meteorological factors, avoiding the potentially unrobust estimates due to small sample size.


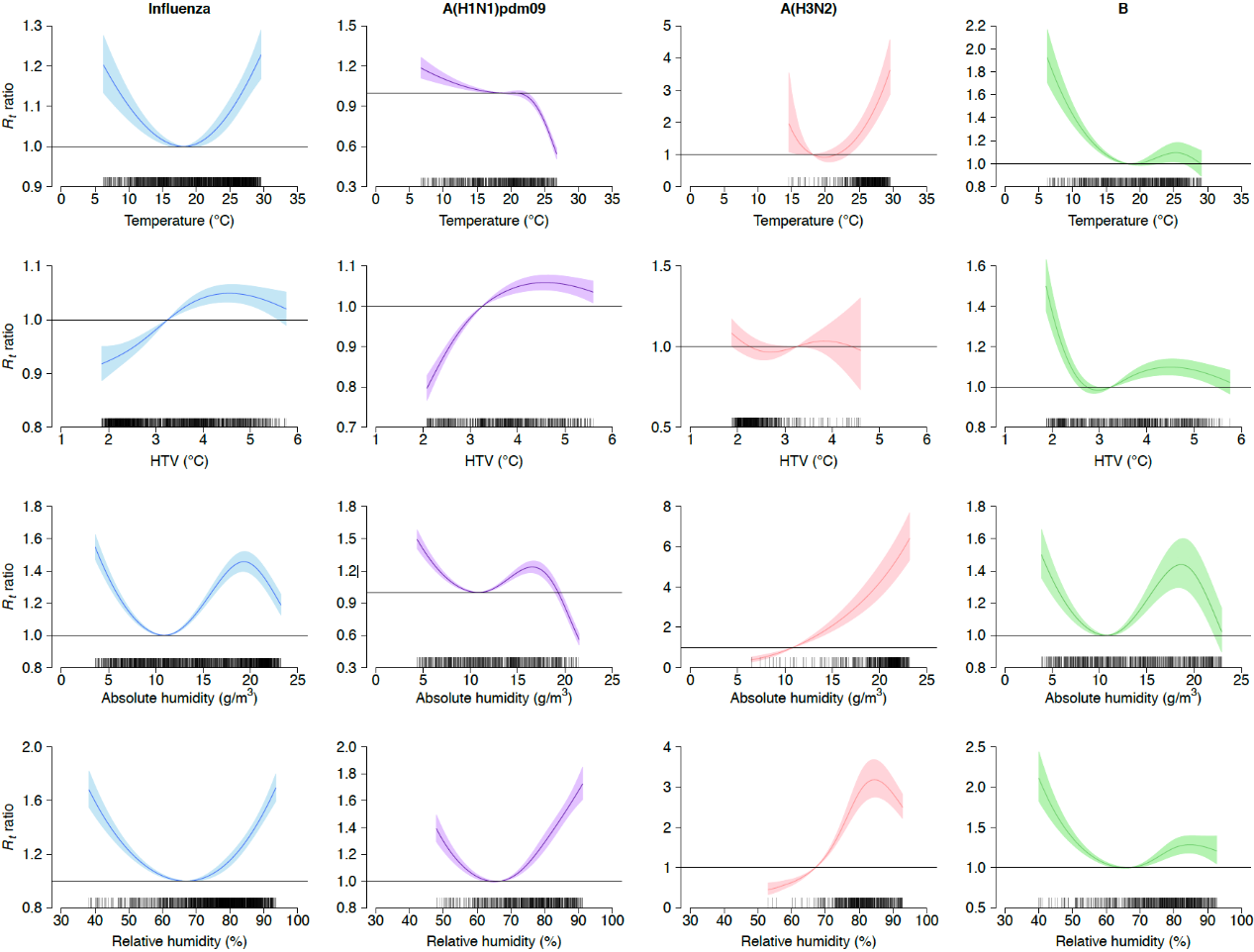


**Additional file 35: Figure S30.** Exposure-response curves of the associations of daily instantaneous effective reproductive number (*R_t_*) with various climatic variables when considering data from maximum ten weeks either side of each epidemic peak. Curves and colored areas represent the point estimates of *R_t_* ratios and the corresponding confidence intervals, respectively. The ticks along the x-axis are observed meteorological data. Horizontal lines indicating *R_t_* ratio = 1 were also plotted. The *R_t_* ratios are the ratio of predicted *R_t_* with respect to reference values for the meteorological factors of mean temperature, hourly temperature variability (HTV), absolute humidity, and relative humidity set to 18.12°C, 3.25°C, 10.88g/m^3^, and 66.99%, respectively. We depicted the associations excluding the ten lowest and the ten largest values of meteorological factors, avoiding the potentially unrobust estimates due to small sample size.

**Additional file 36: Table S4.** Comparisons of studies which assessed the association between meteorological factors and influenza transmissibility

| Study | Study location | Study period | Influenza virus activity proxy used to estimate *R_t_* |
| --- | --- | --- | --- |
| Ali et al. [1] | Nine PLADs of China, including Beijing, Tianjin, Gansu, Shanghai, Zhejiang, Hubei, Jiangxi, Guangdong, Hong Kong | 2005–2016 | ILI+ |
| Lei et al. [6] | Five PLADs of China, including Beijing, Tianjin, Shanghai, Chongqing, Hong Kong | 2013–2019 | ILI+ |
| Zhang et al. [3] | 30 PLADs in Mainland China, excluding Tibet^a^ | 2010–2017 | ILI+ |
| Zhang et al. [4] | Guangzhou | 2005–2021 (including the COVID-19 pandemic) | Reported number of symptomatic influenza cases |
| This study | Guangzhou | 2010–2019 | ILI+ |

Abbreviations: *R_t_*, effective reproductive number; PLADs, provincial-level administrative divisions; COVID-19, Coronavirus Disease 2019.

ILI+ was calculated by multiplying the influenza-like illness (ILI) consultation rate (i.e., the proportion of patients with ILI among the outpatients) by the rate of specimens positive for influenza.

^a^ Tibet was excluded due to the sparse sample intensity.

# References

1. Ali ST, Cowling BJ, Wong JY, Chen D, Shan S, Lau EHY, et al. Influenza seasonality and its environmental driving factors in mainland China and Hong Kong. Sci Total Environ. 2022;818:151724.

2. Johnson KD, Beiglböck M, Eder M, Grass A, Hermisson J, Pammer G, et al. Disease momentum: estimating the reproduction number in the presence of superspreading. Infect Dis Model. 2021;6:706-728.

3. Zhang B, Chen T, Liang S, Shen W, Sun Q, Wang D, Wang G, et al. Subtypes specified environmental dependence of seasonal influenza virus. Sci Total Environ. 2022;852:158525.

4. Zhang R, Lai KY, Liu W, Liu Y, Cai W, Webster C, et al. Association of climatic variables with risk of transmission of influenza in Guangzhou, China, 2005–2021. Int J Hyg Environ Health. 2023;252:114217.

5. Kan, H., Chen, B., Zhao, N., London, S.J., Song, G., Chen, G., et al., 2010 Part I. A time-series study of ambient air pollution and daily mortality in Shanghai, China. In: Public Health and Air Pollution in Asia (PAPA): Coordinated Studies of Short-Term Exposure to Air Pollution and Daily Mortality in Four Cities. HEI Research Report 154. Health Effects Institute, Boston, MA.

6. Lei H, Yang M, Dong Z, Hu K, Chen T, Yang L, et al. Indoor relative humidity shapes influenza seasonality in temperate and subtropical climates in China. Int J Infect Dis. 2023;126:54-63.
